# Zebrafish Whole-Adult-Organism Chemogenomics for Large-Scale Predictive and Discovery Chemical Biology

**DOI:** 10.1371/journal.pgen.1000121

**Published:** 2008-07-11

**Authors:** Siew Hong Lam, Sinnakarupan Mathavan, Yan Tong, Haixia Li, R. Krishna Murthy Karuturi, Yilian Wu, Vinsensius B. Vega, Edison T. Liu, Zhiyuan Gong

**Affiliations:** 1 Department of Biological Sciences, National University of Singapore, Singapore; 2 Genome Institute of Singapore, Agency for Science Technology and Research, Singapore; Stanford University Medical Center, United States of America

## Abstract

The ability to perform large-scale, expression-based chemogenomics on whole adult organisms, as in invertebrate models (worm and fly), is highly desirable for a vertebrate model but its feasibility and potential has not been demonstrated. We performed expression-based chemogenomics on the whole adult organism of a vertebrate model, the zebrafish, and demonstrated its potential for large-scale predictive and discovery chemical biology. Focusing on two classes of compounds with wide implications to human health, polycyclic (halogenated) aromatic hydrocarbons [P(H)AHs] and estrogenic compounds (ECs), we generated robust prediction models that can discriminate compounds of the same class from those of different classes in two large independent experiments. The robust expression signatures led to the identification of biomarkers for potent aryl hydrocarbon receptor (AHR) and estrogen receptor (ER) agonists, respectively, and were validated in multiple targeted tissues. Knowledge-based data mining of human homologs of zebrafish genes revealed highly conserved chemical-induced biological responses/effects, health risks, and novel biological insights associated with AHR and ER that could be inferred to humans. Thus, our study presents an effective, high-throughput strategy of capturing molecular snapshots of chemical-induced biological states of a whole adult vertebrate that provides information on biomarkers of effects, deregulated signaling pathways, and possible affected biological functions, perturbed physiological systems, and increased health risks. These findings place zebrafish in a strategic position to bridge the wide gap between cell-based and rodent models in chemogenomics research and applications, especially in preclinical drug discovery and toxicology.

## Author Summary

To understand chemical-induced biological responses/effects, it is important to have large-scale and rapid capacity to investigate gene expression changes caused by chemical compounds at genome-wide scale in an adult vertebrate model; this capability is essential for drug development and toxicology. Small aquarium fish with vast genomic resources, such as zebrafish, will probably be the only vertebrate models that allow for cost-effective, large-scale, genome-wide determination of gene expression net changes in the entire adult organism in response to a chemical compound. Presently, such a whole adult organism approach is only feasible in invertebrate models such as the worm and fly, and not in rodent models, hence the usefulness of such an approach has not been demonstrated in a vertebrate. By using two classes of chemicals with wide implications to human health, we showed that capturing net changes of gene expression at a genome-wide scale in an entire adult zebrafish is useful for predicting toxicity and chemical classes, for discovering biomarkers and major signaling pathways, as well as for inferring human health risk and new biological insights. Our study provides a new approach for genome-wide investigation of chemical-induced biological responses/effects in a whole adult vertebrate that can benefit the drug discovery process and chemical toxicity testing for environmental health risk inference.

## Introduction

Chemogenomics, application of genomic tools in pharmacology and toxicology, offers a promising approach that will enhance drug discovery (target identification/validation, lead identification, efficacy evaluation) and toxicity assessment [1],[2]. Presently, invertebrates such as the worm *Caenorhabditis elegans* and fly *Drosophila melanogaster*, are the only animal models that have benefited from whole-adult-organism expression chemogenomics [3]–[6]. The benefits of whole-adult-organism chemogenomics would usually translate into large-scale, high-throughput, high-content and cost-effective applications for chemical biology. It is highly desirable that the benefits of whole-adult-organism chemogenomics can be realized in a vertebrate model because of the many biological processes, health risks and diseases that are restricted to a mature vertebrate system including humans. The existing cell-, fly- and worm-based models, while suited for high-throughput chemogenomics, lacked the relevant physiological whole-organism setting of an adult vertebrate. This is especially important in the context of pharmacology and toxicology when many of the potentially targeted organ-systems such as the endocrine, digestive (liver in particular), immune, muscular-skeletal, vasculature, kidney are absent from the existing high-throughput models. In contrast, the rodent models, though providing *in vivo* adult vertebrate data, are not suited for high-throughput applications and are not cost-effective [7], thus creating a bottleneck situation when *in vivo* biological data, especially toxicology, is required for the high number of ‘hits’ generated from *in vitro* screenings or for the many newly emerging industrial compounds and waste that are coming into contact with the public and environment. We propose that whole-adult chemogenomics performed on a small vertebrate such as the zebrafish would be a strategy that is sufficiently high-throughput, cost-effective and would generate high content *in vivo* vertebrate data potentially useful for large-scale screening and toxicity testing purposes.

Conceptually, whole-adult-organism expression chemogenomics would capture the sum-total of the transcriptomic changes in an entire adult organism as a single biological entity responding to exogenous chemical cues. This, however, would have its inherent limitations such as loss of weak signals or signals from smaller tissues and loss of specific location of response, and they may be compounded further by the greater biological complexity in vertebrates compared to invertebrates. Thus, while whole-adult-organism chemogenomics had been shown to be useful in invertebrate models with regard to compound screening [3],[4] and identifying biological processes affected by specific compounds [5],[6], it is not known if chemogenomics data generated from a whole adult vertebrate will be useful. We hypothesized that since strong and well-represented expression signals are likely to be detected in whole-adult-organism chemogenomics, the expression signals that are captured would be robust for predictive chemical biology and for uncovering biology that is widely associated with the chemical-induced responses/effects in the adult vertebrate.

However, the idea of performing high-throughput whole-adult-organism chemogenomics on a vertebrate model was practically not feasible, if not unimaginable, until microarray technology was made available to small aquarium fish such as the zebrafish. The availability of the zebrafish in large numbers, its small size, low husbandry cost, vast genomic resources and its present use in disease modeling [8] and drug screening [9],[10], make the zebrafish ideal for high-throughput whole-adult-organism chemogenomics. Moreover, owing to their close physiological relationship with the environment, fish are highly sensitive to environmental changes particularly exogenous chemical cues; therefore the impact of chemical effects on fish system is more easily defined and readily studied than on terrestrial species [11]. Previously, we and others have shown that zebrafish responded biologically to chemicals, such as small molecules, drugs and environmental toxicants, in a similar manner as mammals [12]–[16]. In this study, we chose P(H)AHs [represented by Benzo[a]pyrene (BAP), 3-Methylcholanthrene (MC), 2,3,7,8-Tetrachlorodibenzodioxin (TCDD)] and ECs [represented by 17-beta estradiol (Es), Diethylstilbestrol (DES), Bisphenol A (Bis)] as model compounds because they represent two classes of chemicals with wide implications to human health. Both P(H)AHs and ECs are potent AHR and ER agonists, respectively, and these receptors are known to cross-talk and they are regulators of important cellular functions that are involved in various biological processes and have been associated with several patho-physiological conditions [17]–[19]. Some of these compounds have been used as drugs or investigated for therapeutic potential [20]–[22]. Moreover, both classes of compounds are also environmental carcinogens and endocrine disruptors that have generated considerable public health concern [23],[24]. By focusing on P(H)AHs and ECs in this study, we performed chemogenomics on whole adult zebrafish and demonstrated that it is good for large-scale predictive chemical biology, for discovering biomarkers and major signaling pathways, as well as useful for human health risk and biological insight inference. Our study placed zebrafish in a strategic position to bridge the gap between *in vitro* cell-based model and *in vivo* rodent model in chemogenomics.

## Results/Discussion

### Robust Predictive Power of Zebrafish Whole-Adult-Organism Chemogenomics

**Figure 1:**
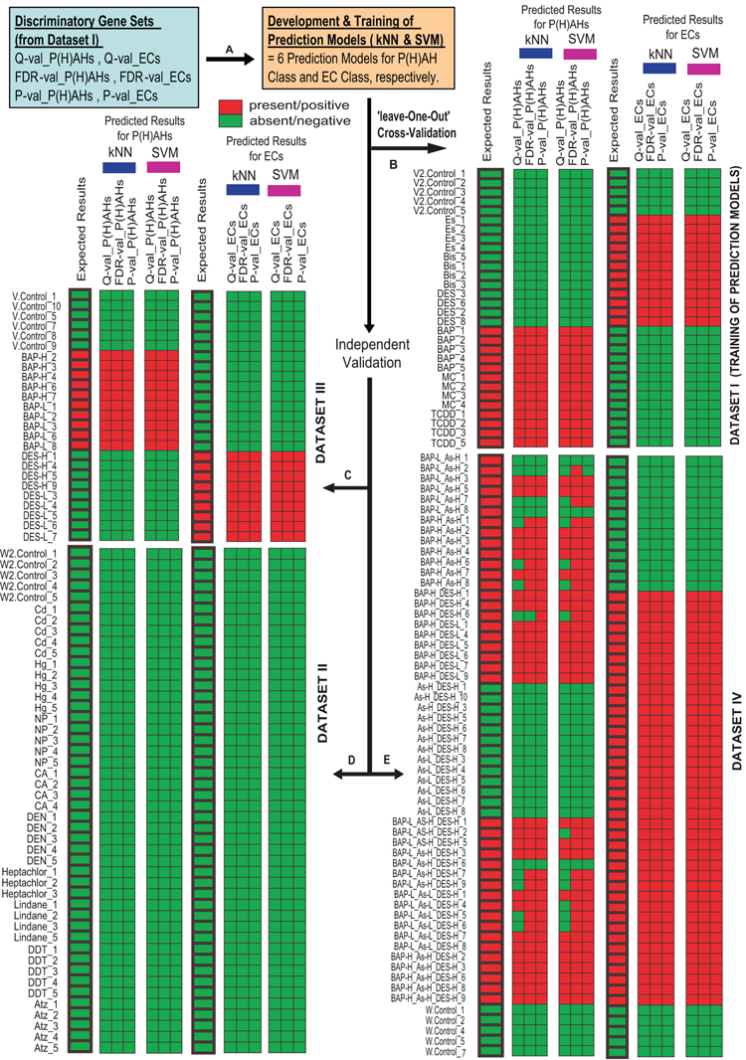
Performance of P(H)AH and EC prediction models trained using discriminatory gene sets from Dataset I. (A) The prediction models were generated by two supervised learning classifiers, k-nearest neighbours (*k*NN) and support vector machine (SVM), using the discriminatory gene sets selected based on different statistical thresholds Q-val, FDR-val and P-val. (B) The prediction models were self-evaluated using ‘leave-one-out’ cross-validation approach followed by independent cross-validation using ‘unseen’ (C) Dataset III, (D) Dataset II and (E) Dataset IV. The labels indicating the actual treatment group for each sample/array are shown on the left side of each dataset panel with the corresponding ‘Expected Results’ columns (bold-lined cells; see Table S1 for detailed information of each label/sample). The remaining columns in groups of three are the ‘Predicted Results’ generated by the prediction models (See Table S2 for information of the gene sets). Red cell indicates presence or positive identification of a class of compound and green cell indicates absence or negative identification of it (see Table S3 for detailed performance of each prediction model).

**Figure 2:**
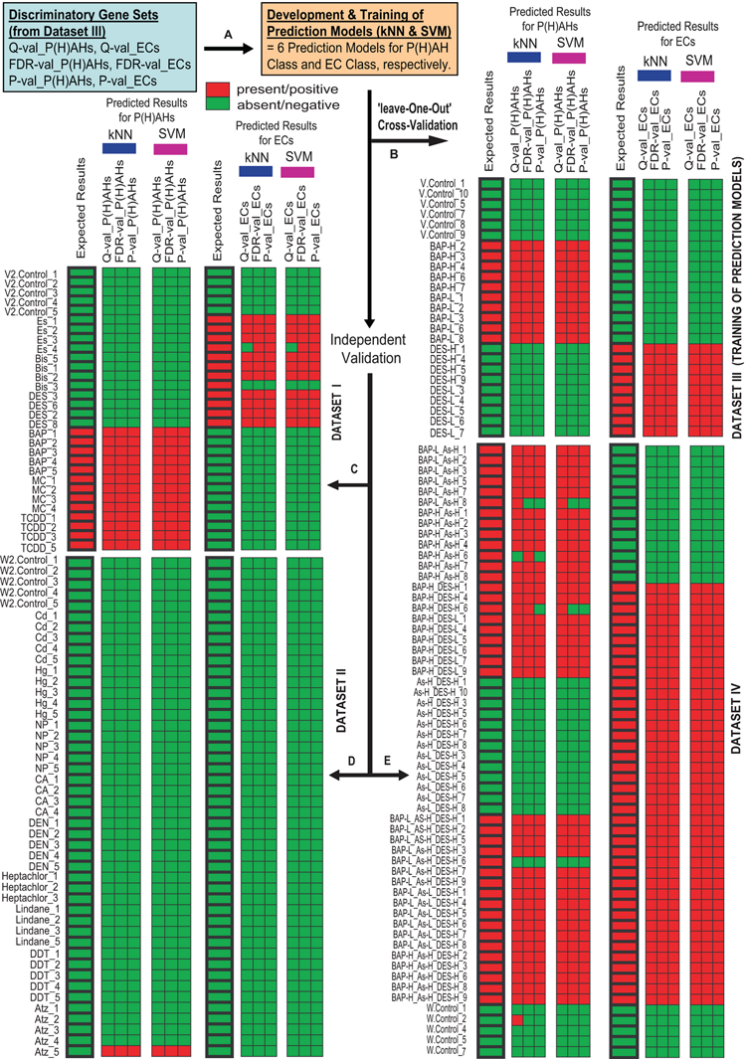
Performance of P(H)AH and EC prediction models trained using discriminatory gene sets from Dataset III. (A) The prediction models were generated by two supervised learning classifiers, k-nearest neighbours (*k*NN) and support vector machine (SVM), using the discriminatory gene sets selected based on different statistical thresholds Q-val, FDR-val and P-val. (B) The prediction models were self-evaluated using ‘leave-one-out’ cross-validation approach followed by independent cross-validation using ‘unseen’ (C) Dataset I, (D) Dataset II and (E) Dataset IV (see Figure 1 legend for remaining description).

We generated 159 samples/arrays involving 28 treatment groups (4–7 replicates in each treatment group) in two experiments (‘A’ and ‘B’) and grouped them into four Datasets: I and II from experiment ‘A’ while III and IV from experiment ‘B’ (See Methods, Table S1 and Figure S1). Experiments ‘A’ and ‘B’ were performed one year apart using different batches of fish, reference RNA, reagents, array prints and experimental designs, which will test the robustness of the prediction models and help in identifying robust biomarkers. We trained six prediction models using Dataset I (Figure 1A and 1B) and validated them independently on Datasets II, III and IV (Figure 1C–1E). The validations were ‘independent’ in the sense that Datasets II, III and IV were totally left out and not used in the training of the prediction models that were tested on. Next, we trained another six prediction models using Dataset III (Figure 2A and 2B) and performed similar independent validation on the ‘unseen’ Datasets I, II and IV (Figure 2C–2E). The prediction models were trained using two supervised learning classifiers, k-nearest neighbours (kNN) and support vector machine (SVM), on selected discriminatory gene sets from Dataset I or III. During the training phase, the supervised learning classifiers used the individual discriminatory gene sets together with their expression data in Dataset I or III to produce respective sets of rules or reference weights that will serve as standards/models for the prediction of ‘unseen’ or unknown samples. The discriminatory gene sets for P(H)AH and EC classes were selected using threshold criteria of Q-value, FDR-value or P-value coupled with fold-difference between treated [combined representative groups of P(H)AHs or ECs] versus control samples in Dataset I or Dataset III (Figure 1A and 2A; details of criteria in Table S2). The different statistical treatments and learning classifiers were used to examine if any idiosyncrasy associated with data processing affected the performances of the prediction models.

First, we evaluated the prediction models using ‘leave-one-out’ cross-validation approach to avoid the statistical problem of over-estimating prediction accuracy that occurs when a model is trained and evaluated with the same samples. In this procedure, one of the 30 or 25 samples from Datasets I or III, respectively, was withheld and the remaining 29 or 24 of the respective samples were used to train a prediction model based on a selected discriminatory gene set to predict the class of the withheld sample. The process was repeated until all 30 or 25 samples were predicted in turn, and all prediction models were tested similarly. We found that the ‘Predicted Results’ for all the samples matched the ‘Expected Results’, hence 100% of the samples were correctly classified by all the prediction models (Figure 1B and 2B). The excellent performances of the prediction models were non-random (Fisher's Exact Test P-value = 8.35×10^−9^−4.89×10^−7^; Table S3), indicating their predictive powers.

To test the robustness of their predictive powers, we sought to validate them independently. In this procedure, we used the 30 samples from Dataset I to train prediction models based on the selected discriminatory gene sets and predict each of the 25 ‘unseen’ samples from Dataset III. Remarkably, the prediction models for P(H)AHs and ECs trained by Dataset I classified 100% of the samples in Dataset III correctly (Figure 1C). We then reversed the order by training the prediction models with the 25 samples from Dataset III and testing them on each of the 30 samples from Dataset I. The prediction models for P(H)AHs trained by Dataset III classified all the samples in Dataset I correctly while the prediction models for ECs performed comparably well though with one or two false negatives (Figure 2C). The findings indicate that the prediction models were remarkably robust because both Datasets I and III were obtained through two separate experiments that contained substantial biological and technical variations.

Next, we tested the specificity (the ability to identify negative cases) of the above prediction models on 9 types of compounds from classes with acute mode-of-action and toxicity effects differing from that of P(H)AHs and ECs. Thus, we tested each of the prediction models generated using the 30 and 25 samples from the respective Datasets I and III, on each of the 46 ‘expected-negative’ samples from Dataset II, independently. As anticipated, with only one exception, all samples were predicted as ‘negative’ matching the ‘Expected Results’ (Figure 1D and 2D). The robust performance of the prediction models generated from Dataset III is noteworthy since both Datasets II and III were derived from different experiments.

We also tested the performance of the prediction models on Dataset IV consisting of 58 samples obtained from fish exposed to multiple chemical mixture of BAP, DES and arsenic (As) at different concentrations and combinations. Arsenic was introduced to increase the complexity of the mixtures and to test if the prediction models could still perform well on samples exposed to mixtures consisting of a chemical not used in the training of the models. The performance of the prediction models for ECs was outstanding as they could classify 100% of the samples correctly notwithstanding the increase complexity that could arise from the mixture of compounds and that Datasets I and IV are from separate experiments (Figure 1E and 2E). The prediction models for P(H)AHs displayed comparable performances in terms of specificity although the sensitivity (ability to identify positive cases) varied from 62.5%–97.5% (Table S3). Notably, prediction models of P(H)AHs trained from Dataset I performed poorer on Dataset IV compared to those trained from Dataset III, suggesting that when mixtures were involved, the performance of the P(H)AH models were affected by the inter-experimental variations. In this case, different statistical approaches can affect prediction performance, but with appropriate statistical tests, it is possible to generate prediction models that are sufficiently robust. This was observed in the case of SVM-trained P(H)AH models using discriminatory gene sets from Dataset I selected based on FDR-val (sensitivity = 92.5%) and P-val (sensitivity = 90.0%) which performed better compared to Q-val (sensitivity = 62.5%) (Table S3).

Taken together, with the exception of Q-val_P(H)AH performance on Dataset IV, all the prediction models performed comparably well, in particular for most of ECs which scored 100% for specificity and sensitivity. The robust ability of the prediction models to discriminate compounds of the same class from those of different classes, even in a mixture of compounds, suggests that zebrafish whole-adult-organism chemogenomics is capturing genes associated with biological functions that are strongly affected by a class of compound. This demonstrates its potential use for compound screening, predictive chemical biology and biomarker discovery. The ability of the prediction models to tolerate a reasonable level of biological, technical and data-processing variations, indicate high amenability to real-life compound screening and predictive applications as such variations will inevitably occur over time, in different laboratories and experimental settings.

### Identification of Potential Biomarker Genes and Signaling Pathways

**Figure 3:**
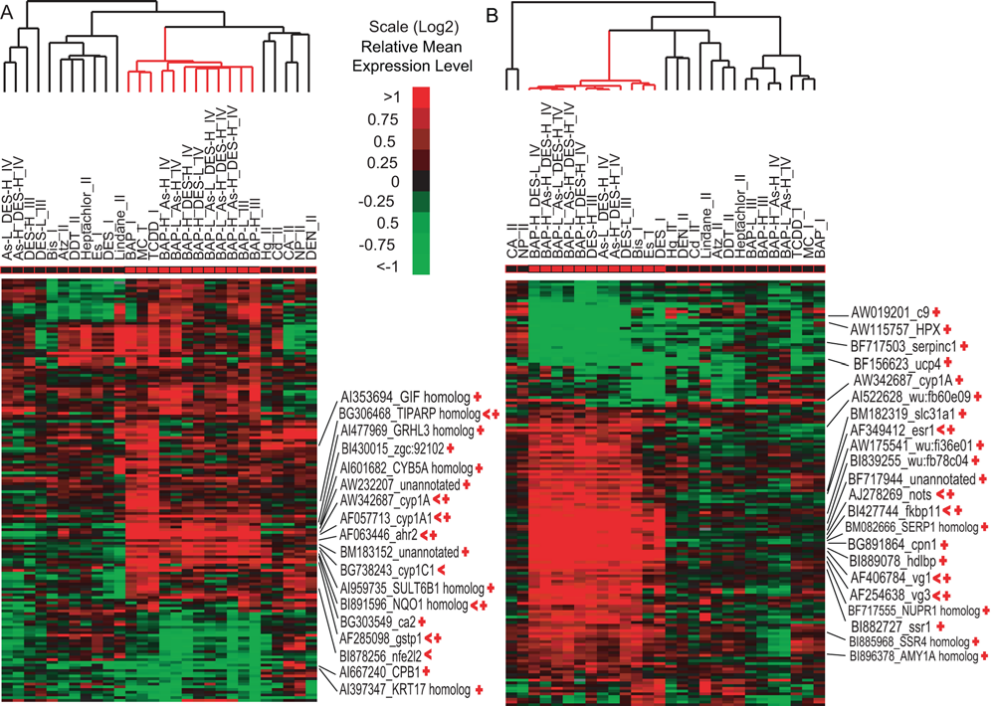
Identification of potential biomarker genes. Two-way hierarchical clustering of the consolidated discriminatory gene sets for (A) P(H)AHs and (B) ECs versus all the treatment groups used in the present study. Each cell represents the relative (log_2_) mean expression level of a gene between the corresponding treatment group and the respective control (vehicle) group [i.e. after subtracting respective control group]. Tight clusters of P(H)AH and EC treatment groups (including mixture groups) are indicated by ‘cluster branches’ in red. Known responsive genes to the respective class of compound/compound are indicated by ‘<’. Genes validated by quantitative real-time PCR are indicated by ‘+’ (see Table S4 for PCR validation information).

Having demonstrated their predictive powers, we used the discriminatory gene sets to identify potential biomarker genes for P(H)AHs and ECs. To do so, we first consolidated the discriminatory gene sets into their corresponding two major groups P(H)AHs and ECs by combining the gene sets within their respective classes (including only genes with unique GenBank Identity and similar mean expression directionality). Then, we examined the consistency of their expression profiles throughout all the 28 treatment groups in this study and validated some of the responsive genes using quantitative real-time PCR. A two-way hierachical clustering showed that the consolidated gene sets were able to cluster tightly the respective treatment groups including those in the mixture groups from the non-P(H)AH (Figure 3A) or non-EC treatment groups (Figure 3B). However, the gene expression profiles formed in the EC gene set (Figure 3B) were more distinct compared to the P(H)AH gene set (Figure 3A). This is due to the large number of genes that are specifically affected by ECs compared to the presence of certain xenobiotic metabolism or stress response-associated genes shared between P(H)AHs and other compounds, as well as the presence of some genes whose expressions are affected by compound mixtures. A closer examination of the genes reveals that *ahr2* and its known regulated/responsive genes [25] such as *cyp1A1, NQO1* homolog, *nfe2l2, TIPARP* homolog, *gstp1, cyp1C1* were among the genes found in a tight cluster that shows similar expression pattern across the P(H)AH treatment groups (Figure 3A). Likewise, *esr1* and its known regulated/responsive genes [26],[27] such as *vg1* and *vg3, nots*, *XBP1* homolog, *NUPRI/P8* homolog were among the genes found in a tight cluster that shows consistent expression pattern across the ECs (Figure 3B). The findings indicate that zebrafish whole-adult-organism chemogenomics is able to capture important genes associated with major signaling pathways such as AHR and ER that are deregulated by the compounds. More importantly, the presence of many unknown genes clustering together with the known P(H)AH- or estrogen-responsive genes, suggests that these are potential novel biomarkers for P(H)AHs and ECs, respectively. The consistent expression patterns across several treatment groups containing P(H)AHs or ECs from two different experiments highlights the robustness of these biomarker genes.

In addition, we have independently validated 27 and 28 genes that were significantly (P-value <0.05) deregulated in the BAP and DES groups, respectively, using quantitative real-time PCR. We later observed that 16 and 21 of these genes were in the consolidated discriminatory gene sets of P(H)AHs and ECs, respectively (Figure 3; Table S4). As expected, there are a large number of significantly deregulated genes that are excluded in the discriminatory gene sets due to non-fulfillment of the stringent selection criteria but they are nevertheless responsive to BAP and/or DES. The good concordance of the microarray and PCR results for these genes gave us the confidence to pin down these biomarkers at the tissue level.

### Biomarker Discovery in Targeted Tissues

To confirm these biomarkers and obtain insights in targeted tissues, we performed a third independent experiment ‘C’ (Table S1) by treating zebrafish with BAP or DES followed by quantitative real-time PCR assay for the previously validated genes (Table S4) in seven tissue types (brain, gills, liver, gut, skin, testis and eyes). Out of the 27 and 28 validated genes for BAP and DES respectively, a total of 81 and 99 positive hits [significant gene deregulation (T-Test P-value <0.05)] were detected among the seven tissues (Table 1 and 2; Figure S2). About 37.0% (10/27) and 53.6% (15/28) of the validated genes in BAP and DES, respectively, were deregulated in 4–7 (>50%) of the selected tissues making them excellent biomarker genes for the respective class of compounds (Table 1 and 2). Among these 10 and 15 biomarkers for BAP and DES, respectively, 3 are well-known P(H)AH-responsive genes (*ahr2*, *cyp1A1*, and *TIPARP* homolog) and another 3 are well-known EC-responsive genes (*esr1, vg*1, and *vg3*), while the remainder 19 are potentially novel as their responsiveness to these compounds are relatively unknown/unreported especially within multiple tissue types. The P(H)AH biomarker genes are mainly associated with xenobiotic metabolism while the EC biomarker genes are mainly associated with molecular transport, metabolism and blood factors. Notably, about 90% (22/25) of these biomarker genes were found in the consolidated discriminatory gene sets. The remaining genes that were deregulated in 1-3 tissues may be useful biomarkers for tissue-specific analysis. Less than 10% of the validated genes in the tissues were found to be inconsistent with the whole fish data and this could be due to having not selected the appropriate responsive tissues or biological variations and cumulative effects of several tissues in the whole fish.

**Table 1:** Real-Time PCR validated genes for identification of biomarkers in selected tissue samples from fish treated with BAP (250ug/L) for 72 hours.

			Tissue Types (Log2 Fold Difference Treated vs. Normal) ***							Number of Positive Tissue
GenBank Acc	Description	Whole Fish	Brain	Gill	Liver	Gut	Skin	Testis	Eye	
AW342687	Cytochrome P450,1A **	5.44	6.04	7.20	3.75	5.12	6.91	5.55	6.51	7#
AF057713	Cytochrome P450,1A1 **	3.97	3.62	5.61	2.47	3.51	3.90	3.47	3.75	7#
BG306468	TIPARP homolog **	1.97	0.57	1.64	2.03	1.69	NS	1.02	1.40	6#
AI959735	SULT6B1 homolog	2.96	NS	2.29	NS	1.59	1.67	1.98	1.42	5#
BM183152	CDNA clone IMAGE:7177046	2.82	0.95	3.73	NS	NS	2.41	1.01	1.02	5#
AW232207	Transcribed locus	3.17	NS	NS	1.76	NS	3.24	1.16	2.58	4#
BI430015	Zgc:92102	2.36	1.26	1.13	NS	NS	1.59	NS	1.14	4#
AI601682	CYB5A homolog	1.64	NS	3.33	NS	NS	3.10	1.42	1.76	4#
AF063446	Aryl hydrocarbon receptor 2 **	1.41	0.81	NS	1.06	NS	NS	1.70	0.82	4#
BI891596	NQO1 homolog **	1.49	NS	1.49	NS	NS	1.00	NS	1.02	3
AF285098	Glutathione S-transferase pi **	1.00	NS	2.00	NS	1.09	1.15	NS	NS	3
BM025955 *	Transcribed locus	3.23	NS	NS	NS	NS	1.40	NS	1.40	2
BG303549	Carbonic anhydrase II	2.06	NS	NS	NS	NS	2.33	NS	−0.64	2
AW232794 *	Sortilin 1	1.31	0.44	NS	NS	NS	0.52	NS	NS	2
AI477969	GRHL3 homolog	0.96	NS	NS	NS	NS	0.80	1.59	NS	2
BI878941 *	Zgc:77849	1.15	NS	NS	1.00	NS	NS	NS	NS	1
BG304178 *	Transcribed locus	0.49	NS	NS	NS	NS	NS	0.65	NS	1
AI353694	GIF homolog ˆ	1.63	NS	NS	NS	NS	NS	NS	−1.23	1
AI793886 *	Wu:fc55f04 ˆ	0.34	NS	NS	NS	NS	NS	NS	NS	0
AI397347	KRT17 homolog	−0.81	−0.50	NS	NS	−0.65	−0.82	NS	−1.20	4#
AW018635 *	Transforming growth factor, beta-induced	−0.71	−0.48	−0.81	−1.53	NS	NS	NS	NS	3
BG304220 *	Wu:fl33b06	−1.18	NS	1.05	−0.91	NS	NS	NS	−0.68	3
AF295407 *	Alcohol dehydrogenase 8a	−1.57	NS	NS	−1.23	NS	NS	−1.29	−1.12	3
AW421939 *	Hm:zeh1207	−1.07	NS	NS	−1.00	−0.62	NS	NS	NS	2
AW233556 *	Wu:fj37e01	−0.33	NS	NS	NS	NS	NS	NS	−0.35	1
AF064835 *	Eukaryotic translation elongation factor 2 *	−0.41	NS	NS	−0.43	NS	NS	NS	NS	1
AI667240	Carboxypeptidase B1	−1.03	NS	NS	NS	−0.94	NS	NS	NS	1
	Total Number of Positive Hits:		9	11	11	8	14	11	17	31
	Percentage of Positive Hits:		29.03%	35.48%	35.48%	25.81%	45.16%	35.48%	54.84%	

*: Not in the Discriminatory Gene Sets.

**: Known P(H)AHs-responsive genes.

***: All values shown are significant (P-value<0.05, n = 4) by heteroscedastic T-Test when compared to their respective control group.

ˆ Inconsistent with whole fish real-time PCR validated data.

NS: Not Significant (*P*-value>0.05).

# Confirmed as robust biomarker genes based on their significant deregulation in >50% tissues examined.

**Table 2:** Real-Time PCR of validated genes for identification of biomarkers in selected tissue samples from fish treated with DES (5ug/L) for 72 hours.

			Tissue Types (Log2 Fold Difference Treated vs. Normal) ***							Number of Positive Tissue
GenBank Acc	Description	Whole Fish	Brain	Gill	Liver	Gut	Skin	Testis	Eye	
AF406784	vitellogenin 1 **	12.30	NS	3.69	12.94	12.14	9.95	7.71	3.73	6#
AF254638	Vitellogenin 3, phosvitinless **	12.87	NS	NS	10.62	9.88	7.33	5.85	2.79	5#
AF349412	Estrogen receptor 1 **	4.60	−1.00	1.08	3.02	3.49	1.81	NS	NS	5#
BI889078	High density lipoprotein-binding protein	1.31	NS	NS	4.51	0.80	0.86	0.93	−0.63	5#
BI882727	Signal sequence receptor, alpha	1.19	NS	−0.79	4.13	0.84	0.83	NS	−0.35	5#
AW175541	Wu:fi36e01	2.29	NS	−1.50	5.84	NS	1.49	NS	−1.04	4#
BI896378	Zgc:66313	1.65	NS	−1.27	2.90	1.41	3.07	NS	NS	4#
BM182319	Solute carrier family 31, member 1	1.40	NS	NS	3.99	NS	1.33	1.01	−0.37	4#
BF717944	Transcribed locus	4.74	NS	NS	9.35	4.68	3.89	NS	NS	3
BG891864	Carboxypeptidase N, polypeptide 1	2.88	NS	NS	4.86	1.93	2.42	NS	NS	3
BM082666	Zgc:92744	1.97	NS	NS	3.92	0.67	1.40	NS	NS	3
BF717555	nuclear protein 1	3.52	NS	NS	4.69	NS	1.13	NS	NS	2
BI427744	FK506 binding protein 11 **	3.31	NS	NS	8.33	NS	2.61	NS	NS	2
AI522628	Wu:fb60e09	1.74	NS	NS	3.39	NS	1.82	NS	NS	2
BI878941 *	Zgc:77849	0.73	NS	NS	1.64	NS	0.77	NS	NS	2
BI885968	Signal sequence receptor, delta	1.37	NS	NS	3.19	NS	NS	NS	NS	1
BI981380 *	Wu:fj36g07 ˆ	0.38	−2.76	NS	NS	NS	NS	NS	−1.87	2
BI839255	Wu:fb78c04 ˆ	0.59	−1.90	NS	NS	NS	NS	NS	NS	1
AW115757	Hemopexin	−3.23	NS	−2.06	−7.68	−8.12	−3.67	−5.76	−1.45	6#
BF717503	Serine/cysteine proteinase inhibitor C1	−0.89	−1.13	−1.15	−5.39	−1.93	NS	−3.35	NS	5#
AW342687	Cytochrome P450,1A	−0.95	NS	−1.00	−1.95	−1.32	−2.21	NS	−0.71	5#
AW019124 *	Alanine-glyoxylate aminotransferase	−1.47	NS	−0.80	−0.85	−1.82	NS	NS	−1.01	4#
BG985468 *	Fructose-1,6-bisphosphatase 1	−2.09	NS	−2.01	−3.99	−1.30	NS	−5.09	NS	4#
AI722510 *	MGC:103610 IMAGE:7250917	−2.11	NS	NS	−1.76	−3.31	NS	−1.65	−2.67	4#
AW019201	Complement Component 9	−2.35	NS	NS	−3.21	−4.78	NS	−2.84	−1.92	4#
AF057713 *	Cytochrome P450,1A1	−0.65	NS	NS	−1.31	−1.06	NS	−1.66	NS	3
BF156623	Uncoupling protein 4	−1.83	NS	NS	−5.17	−1.75	NS	NS	−2.32	3
AW421213 *	Phenylalanine hydroxylase	−0.68	NS	NS	−0.91	−2.62	NS	NS	NS	2
	Total Number of Positive Hits:		4	10	26	19	17	10	13	29
	Percentage of Positive Hits:		13.79%	34.48%	89.66%	65.52%	58.62%	34.48%	44.83%	

*: Not in the Discriminatory Gene Sets.

**: Known Estrogen-responsive genes.

***: All values shown are significant (P-value<0.05, n = 4) by heteroscedastic T-Test when compared to their respective control group.

ˆ Inconsistent with whole fish real-time PCR validated data.

NS: Not Significant (*P*-value>0.05).

# Confirmed as robust biomarker genes based on their significant deregulation in >50% tissues examined.

Among the selected tissues, eye and skin had the most BAP-responsive genes (63.0% and 51.9% of the validated genes, respectively) followed by gill, liver and testis (40.7% each tissue); these tissues yielded 79.1% of the total positive hits (Table 1; Figure S2). The findings are consistent with mammalian data as these organs (eye, skin, lung, liver and testis) are also known P(H)AH-targeted tissue in mammals [24],[28],[29]. As for tissues with the most DES-responsive genes, liver (92.9%) followed by gut (67.9%) and skin (60.7%) contributed to 62.6% of the total positive hits (Figure S2). The identification of these non-classical estrogen-targeted tissues is consistent with our previous data [30] as well as mammalian data [19],[31]. Activation of AHR or ER signaling pathways, as suggested by up-regulation of known responsive genes, was observed in many of these tissues. Interestingly, *cyp1A* which was up-regulated by BAP in all 7 tissues, was down-regulated by DES in 5 tissues, suggesting occurrence of similar inhibitory cross-talk between AHR and ER reported in mammalian cells [17],[18],[32]. Taken together, the findings show that the zebrafish shares similar biological responses in terms of molecules, signaling pathways and targeted tissues with mammalian system and is therefore a useful model for inference of chemical biology and health-risk inference in humans.

### Biological Function and Human Health-Risk Inferences

**Figure 4:**
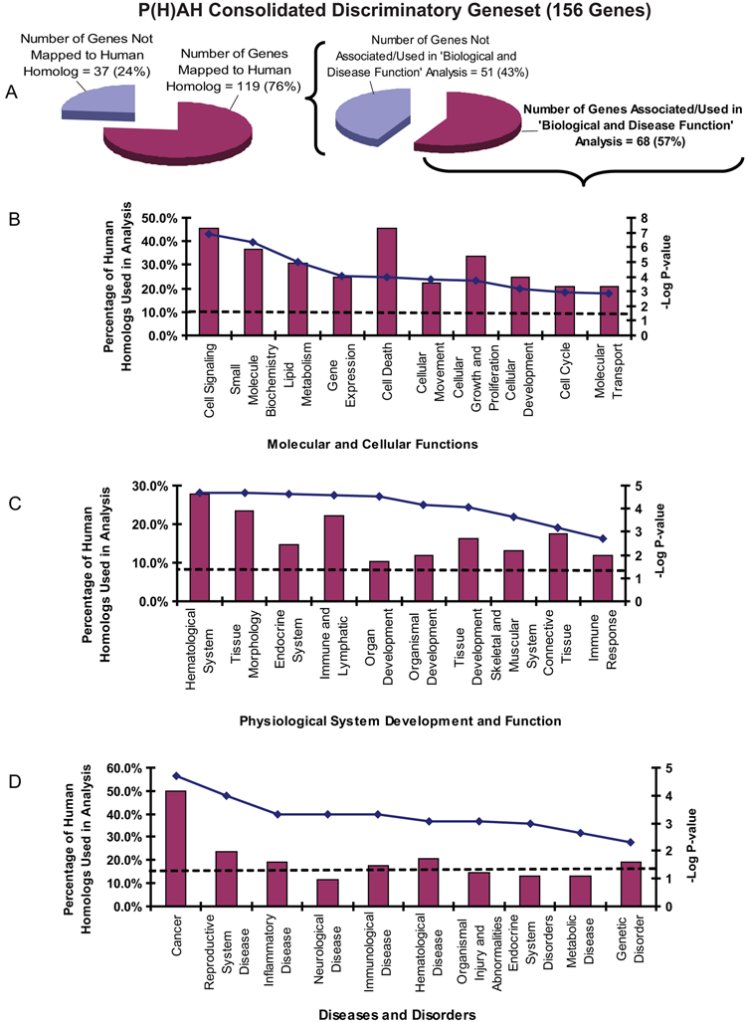
Human homolog mapping, biological function and health-risk inferences for P(H)AH consolidated gene set. (A) 119 genes (among 156) were mapped to human homologs (with human Unigene identity) and used for Ingenuity knowledge-based data mining. Only 68 (57%) of the human homologs from the P(H)AH gene set were found to be associated with certain biological data in Ingenuity and used for subsequent analysis which generated three categories of data: (B) Molecular and Cellular Functions, (C) Physiological System Development and Function, and (D) Diseases and Disorders. Histograms are read with reference to ‘Percentage of Human Homologs Used in Analysis’ axis while solid and dashed lines are read with reference to ‘-Log P-value’ axis. ‘Percentage of Human Homologs Used in Analysis' refers to the percentage of the total 68 human homologs used in the analysis. Solid line represents the inverse logarithm (base 10) of the P-value for each group of biological association [greater –Log (P-value) correlates with greater statistical significance], while the dashed line represents the significant threshold where the P-value = 0.05. Only the top 10 features with the highest percentage of human homologs involved and which are statistically significant are shown (see Table S6 for more information).

**Figure 5:**
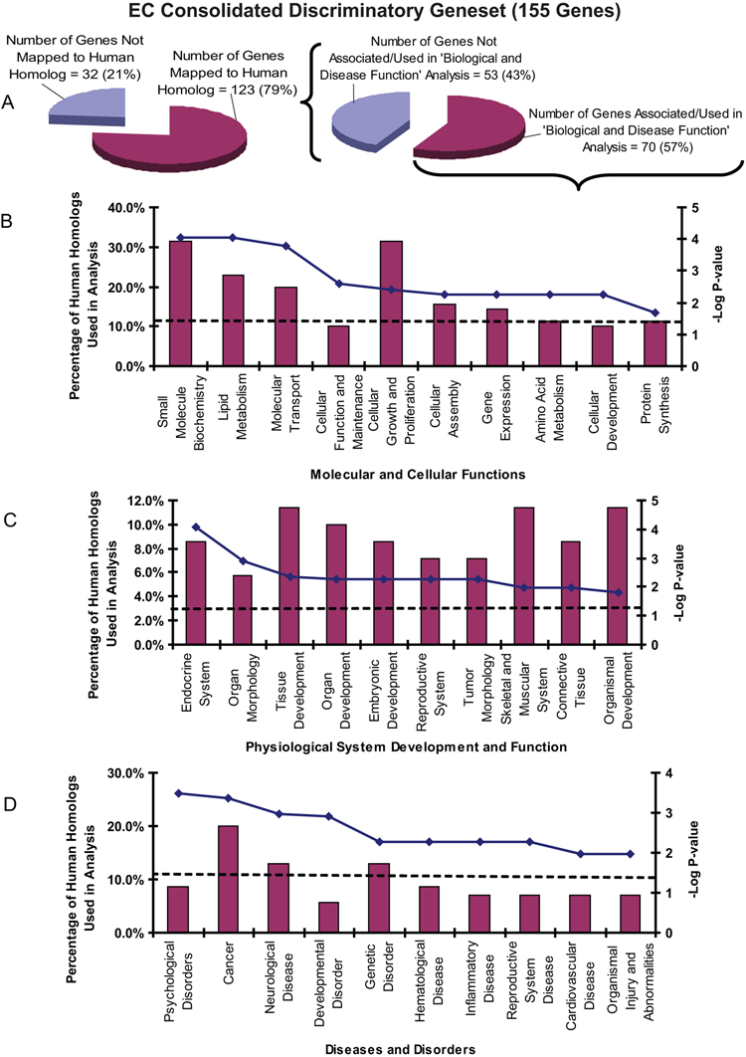
Human homolog mapping, biological function and health-risk inferences for EC consolidated gene set. (A) 123 genes (among 155) were mapped to human homologs (with human Unigene identity) and used for Ingenuity knowledge-based data mining. Only 70 (57%) of the human homologs from the EC gene set were found to be associated with biological data in Ingenuity and used for subsequent analysis which generated three categories of data: (B) Molecular and Cellular Functions, (C) Physiological System Development and Function, and (D) Diseases and Disorders (see Figure 4 legend for remaining description).

To evaluate the potential for extracting biological insights and health-risk inferences in humans, we mapped the consolidated discriminatory gene sets for P(H)AHs and ECs to available corresponding human homologs as previously described [14] and used them for knowledge-based data mining via Ingenuity Pathway Analysis (IPA) software (Figure 4A and 5A). Remarkably, the analysis of the human homologs from the consolidated P(H)AH and EC gene sets listed many affected molecular and cellular functions, perturbed physiological systems and human diseases/disorders that are known to be associated with these compounds [19], [23], [24], [28]–[31] (Figures 4 and 5; Tables S5 and S6). These also include canonical signaling pathways such as xenobiotic mechanism signaling (AHR, CYP1A, CYP1B1, CYP2C19, GSTP1, HSP90A, NFE2L2, NOS2A, NQO1, SULT2B1), ERK-MAPK signaling (EFL3, RAC1, STAT1, RPS6KA1) and PPARa/RXRa activation (CYP2C19, HSP90A, LPL, FABP1, NOS2A, ALAS1)for P(H)AH gene set and lipid metabolism (ACSL4, CYP1A1, CYP2C19, CYP51A1), protein ubiquitination pathway (PSMB6, PSMC2, PSMC5/SUG1, PSMC6, PSMD13) and coagulation cascade (FGB, SERPINA1, SERPINC1) for EC gene set. A correlation can be observed between these biological associations suggesting that prolonged or substantial perturbation of these biological functions (Figures 4B and 5B) and physiological systems (Figures 4C and 5C) by a compound would increase the susceptibility/risk of certain diseases/disorders (Figures 4D and 5D). Significantly, cancer was listed among the top most (Fisher Exact Test P-value = 1.98×10^−5^−4.76×10^−2^) associated disease as most P(H)AHs and some ECs are potent carcinogens. In addition, reproductive system disease, inflammatory disease, hematological disease and neurological disease were significantly associated with both P(H)AHs and ECs as the two classes of compounds are known to affect molecules and functions involved with reproductive system, inflammation, blood and nervous system (Figures 4C–D and 5C–D). Interestingly, the association of psychological disorders with ECs were also significant (P-value = 3.34×10^−4^−3.72×10^−2^; Table S6). While it is well-known that estrogen affects mental health [33], the grouping of ESR1 with GPX4, PSMC6, DIABLO, FBXO9, XBP1 homologs which have been associated with bipolar disorder [34],[35] suggests that these molecules may also play a role in estrogen-related psychological disorders (Table S6). Several of these patho-physiological systems indicated to be affected by the compounds (Table S5 and S6), such as reproductive, respiratory, dermatological/connective tissue, digestive/metabolic, nervous/neurological and visual/ophthalmic, corroborated with our multiple targeted-tissue analysis which showed that many of the biomarkers were deregulated in these tissues (Tables 1 and 2; Figure S2), suggesting that they are good ‘biomarkers of effect’.

Thus, a whole-adult-organism representation fits well for human health-risk inferences as the biological information are obtained, not from single tissue but multiple tissues interacting in a complex biological system where diseases/disorders usually develop or off-target effects occur. Incidentally, while zebrafish has always been used to model after human diseases [8], here we demonstrate the potential of zebrafish for predicting disease susceptibility or health risk associated with the exposure to a compound, and this in turn can further help to develop chemical-induced zebrafish models of human diseases.

### Novel Biological Insights Inferences

**Figure 6:**
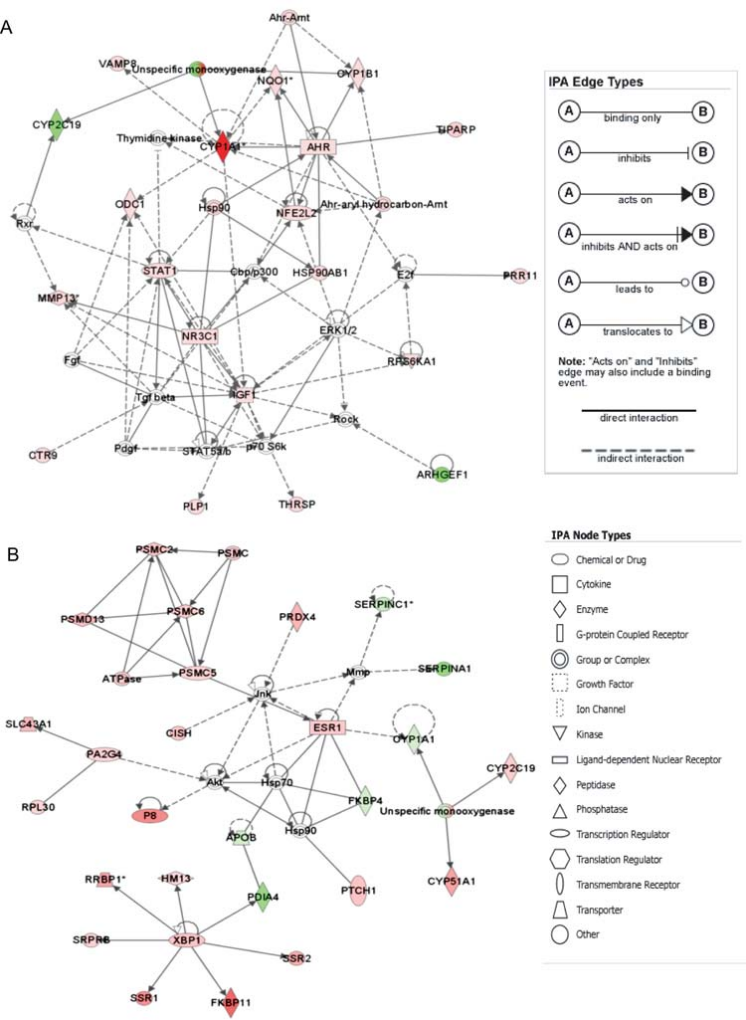
Gene network analysis for biological insights inference. Top networks for (A) P(H)AHs and (B) ECs were generated by Ingenuity Pathway Analysis (IPA) software. Up- and down-regulated genes are indicated in red and green symbols, respectively. Non-colored genes are not in the discriminatory gene sets but are associated with the deregulated genes and are introduced by the software to link up the network.

A closer examination of the top connected networks generated by IPA using P(H)AH and EC datasets revealed interesting biological insights (Figure 6). In the P(H)AH network (Figure 6A **;** P-value = 1.00×10^−39^) the well-established xenobiotic-responsive molecules (AHR, CYP1A1, CYP1B1, CYP2C19, HSP90AB1, NFE2L2, NQO1, TIPARP) were linked with key signaling molecules such as NR3C1/GR, STAT1 and IGF1 providing new insights into alternative mechanisms of how P(H)AHs could exert adverse effects resulting in various pathological conditions. Notably in Table S5, 6 categories of P(H)AH-related diseases/disorders were found to be associated with AHR, NR3C1/GR, STAT1 and IGF1, while all 24 categories of the related diseases/disorders were associated with at least one of the four molecules. Interestingly, it was only recently that stronger evidence of cross-talk between AHR and NR3C1/GR are emerging [36],[37]. Our analysis suggests that HSP90 [38] may be one of the mediators, as also observed in the relatively more studied AHR-ER cross-talk [39]. As both NR3C1/GR and STAT1 are known regulators of inflammatory and immune response [40], respectively, the AHR-NR3C1/GR cross-talk may be another pathway that could contribute to the known immuno-toxicity effects of P(H)AHs [41]. The opposing direction of expression for CYP1A1 and CYP2C19, as displayed in both P(H)AHs and ECs networks (Figure 6), are evidence of inhibitory cross-talk between AHR and ER. While CYP1A1 is a known targeted molecule of this inhibitory AHR-ER cross-talk [32], this is the first time a member of the CYP2 family that includes important drug metabolizing enzymes is implicated and its regulation appeared opposite to CYP1A1. Apart from the xenobiotic-responsive molecules, the ECs network (Figure 6B **;** P-value = 1.00×10^−57^) linked clusters of molecules associated with proteasome-mediated degradation (PSMC2, PSMC5/SUG1, PSMC6, PSMD13), endoplasmic-reticulum stress response (FKBP11, HM13, RRBP1, PDIA4, SRPRB, SSR1, SSR2, XBP1), and cell cycle/death (CISH, FKBP4, P8, PA2G4, PTCH1), providing insights into cellular homeostasis and pathology associated with ECs and ER [19],[42]. The linking of the 5 known estrogen-responsive transcription factors PSMC5/SUG1, XBP1, P8, PA2G4 and ESR1 suggests that, under the influence of ECs, these transcription factors play important roles in mediating endoplasmic-reticulum stress response, proteasome-mediated degradation and cell cycle/death, thus offering new insights into the regulation of ‘unfolded protein response’ which has been intensely studied due to its association with diseases, drug resistance and its potential as therapeutic targets [43]–[45]. While further investigation is warranted, the analysis demonstrate the discovery potential of zebrafish whole-adult-chemogenomics and serve the purpose of alerting the researcher of the potential molecular interactions and effects induced by the compounds at the early drug discovery stage.

In summary, we have demonstrated that zebrafish whole-adult-organism chemogenomics is practical and effective for large-scale predictive and discovery chemical biology. Specifically, we have generated robust prediction models, identified and validated biomarker genes in multiple targeted tissues, identified important signaling pathways and biological functions as well as inferred human health risks and biological insights for both P(H)AHs and ECs. As strong and well-represented expression signals are likely to be captured, this approach is valuable for acquiring a molecular snapshot of the chemical-induced biological state of an adult vertebrate, which includes biomarkers of effects, deregulated signaling pathways, as well as possible affected biological functions, perturbed physiological systems and increased health risks. Moreover, this approach allows for rapid sampling in large-scale experiments, abundant sample materials for assays (does not require pooling or amplification of samples) and easy scaling-up of experiments, hence affords greater statistical power for data analysis. These are essentials for successful high-throughput genomics applications and for building up large database for predictive chemical biology [2]. The zebrafish is more cost-effective than the rodent model for *in vivo* toxicology [12],[13] and there had been proposals and successful attempts of using chemical screens and toxicity testing in whole-adult zebrafish as there are biological processes and diseases that are mainly associated with adults [9],[46]. Therefore, with zebrafish, cost-effective *in vivo* adult vertebrate chemogenomics can be performed earlier in the drug discovery process and for industrial/environmental toxicology, where high resolution tissue-specific data is yet required but robust and informative *in vivo* toxicological data is deemed valuable. This will enable researchers to better understand the potential liabilities of new compounds before advancing them to clinical test and may help shift attrition upstream [2],[47],[48] or before allowing contact with the public or releasing them to the environment. This present study has provided a new strategy for genome-wide investigation of chemical-induced biological responses/effects in a whole-adult vertebrate model; where previously such whole-adult-organism chemogenomics approach were thought only feasible in invertebrate models. The realization of its potential can benefit the drug discovery process and toxicology, in particular chemical toxicity testing for environmental health-risk inference.

## Materials and Methods

### Chemical Exposure and Fish Samples

Three independent experiments (‘A’, ‘B’ and ‘C’) were performed in this study where different batches of adult male zebrafish were exposed to various chemical compounds at different concentrations (Table S1). The selected chemicals represent compounds with toxicological interests and/or environmental-health importance, and the concentrations used were based on available published data or our preliminary acute toxicity exposure experiments conducted for the compounds. Experimental procedures were performed within the guidelines of National University of Singapore's Institutional Animal Care and Use Committee (NUS-IACUC). The fish were immersed in the chemical solutions for 96 hours [experiment ‘A’] or 72 hours [experiment ‘B’ and ‘C’] at a density of 1 fish/200 ml at 27±2°C in a static condition. Control fish were kept in vehicle solution or water under similar condition. Chemical solutions and water were changed daily. At the end of experiment, individual whole fish were snap-frozen and pounded to powder in liquid nitrogen for subsequent total RNA extraction using Trizol reagent (Invitrogen, USA) protocol. For experiment ‘C’, specific tissues were snap-frozen in liquid nitrogen for total RNA extraction using RNeasy Mini Kit (QIAGEN, Germany) followed by DNAseI treatment and heat inactivation. Reference RNA was obtained by pooling total RNA extracted from male and female zebrafish. The integrity of RNA samples was verified by gel electrophoresis, and the concentrations were determined by UV spectrophotometer.

### Zebrafish Oligonucleotide Microarray and Hybridization

The oligonucleotide probes for this array were designed by Compugen (USA) and synthesized by Sigma Genesis (USA). The arrays contained 16,416 oligonucleotide probes. The probes were resuspended in 3× SSC at 20 µM concentration and spotted onto in-house poly-L-lysine-coated microscope slides using a custom-built DNA microarrayer in the Genome Institute of Singapore (GIS). The arrays were spotted and quality controlled essentially as described by Eisen and Brown [49].

For fluorescence labeling of cDNAs, 20 µg of total RNA from the reference and sample RNAs were reverse transcribed in the presence of dNTPs mixed with Aminoallyl-dUTP (Sigma, USA) followed by coupling with mono-functional NHS-ester Cy3 and Cy5 dyes (Amersham, USA), respectively. A common reference design is used where equal amount of RNA samples from control and chemical-treated group were labeled with Cy5 and the same amount of common pooled reference RNA is labeled with Cy3. For each array, the Cy5-labeled samples from either the control or chemical-treated group was co-hybridized with the Cy3-labeled common reference. Thus, the respective paired Cy5- and Cy3-labeled cDNAs were pooled, concentrated, and resuspended in DIG EasyHyb (Roche Applied Science) buffer for hybridization at 42°C for 16 h in a hybridization chamber (Gene Machines). After hybridization, the slides were washed in a series of washing solutions (2× SSC with 0.1% SDS, 1× SSC with 0.1% SDS, 0.2× SSC and 0.05× SSC; 30 sec each), dried using low-speed centrifugation, and scanned for fluorescence detection.

### Data Acquisition

The arrays were scanned using the GenePix 4000B microarray scanner (Axon Instruments, USA) and the generated images with their fluorescence signal intensities were analyzed using GenePix Pro 4.0 image analysis software (Axon Instruments, USA). All the arrays gave a mean signal to background ratio more than 5 and had >90% of the gene features that gave a measurable signal. Only gene features that were not flagged were extracted and subjected to Lowess normalization for further analyses. The microarray raw data have been formatted to be compliant with MIAME standard.

### Statistical Procedures for Microarray Data

Statistical comparison of the relative mean expression level for each gene between test groups [combined representative groups of P(H)AHs or ECs] and their respective control groups from Dataset I or III were performed using Student's T-test and Significance Analysis of Microarray [50] (SAM) yielding respective *P-* and *Q-*values for each gene. The resulting P-values were further adjusted for Benjamini and Hochberg False Discovery Rate (FDR). The discriminatory gene sets were selected based on statistical threshold indicated by Q-value, FDR-value and P-value coupled with 1.5 fold-difference between treated versus control samples (Figure S1 and Table S2). The discriminatory gene sets together with their expression data in Dataset I or III were used to train two supervised learning classifiers, kNN and SVM, which generated prediction models for P(H)AHs class and ECs class. The procedure includes a training phase and a testing phase. In the training phase, the discriminatory gene sets together with their expression data from Dataset I or III were used as inputs to produce a set of rules or reference weights as standards/models for the testing phase. A ‘leave-one-out’ cross-validation is usually incorporated to validate the goodness of the model to avoid ‘over-fitting’ it (i.e. only good for predicting the training dataset but not sufficiently generalize to work well on other new and unknown datasets). The testing phase uses the standards created during ‘training’ to assign a discriminator score to each unseen (not used in the training) or unseen sample. Based on this score each sample is placed ‘into’ or ‘out of’ the class and their performances in terms of prediction specificity and sensitivity were determined (Figure 1 and 2; Table S3). Fisher's Exact Test was further used to determine that the performances of the prediction models were non-random.

### Quantitative Real-Time PCR

Equal amounts of total RNA samples from reference, control and test groups were reverse transcribed to cDNA. The cDNA samples were used for quantitative real-time PCR analysis, performed using the Lightcycler system (Roche Applied Science) with Lightcycler-FastStart DNA Master SYBR Green 1 (Roche Applied Science) according to the manufacturer's instructions. Statistical comparison of the relative mean expression level for each gene between test and control groups was performed using Student's T-test and P-value<0.05 is considered significant.

### Human Health-Risk and Biological Insights Inference via Knowledge-Based Data Mining

To evaluate the potential for human health-risk inference, the zebrafish genes were mapped to their corresponding human homologs using the GIS Zebrafish Microarray Annotation Database (http://giscompute.gis.a-star.edu.sg/govind/unigene_db/) http://giscompute.gis.a-star.edu.sg/govind/zebrafish/version2/as previously described [14]. The human homologs of the zebrafish genes from the consolidated gene sets P(H)AHs and ECs were used to mine the human database via Ingenuity Pathways Knowledge Base software (www.ingenuity.com). The ‘Biological and Disease Function Analysis’ was performed to identify biological functions and systems as well as diseases/disorders that were significantly associated with the gene sets. Fisher's Exact test was used to calculate P-values in determining the probability that each biological function, system and disease assigned to that data set is due to chance alone. P-value<0.05 is considered significant by the algorithm. Networks are generated from the available human homologs mapped from the discriminatory gene sets, by maximizing the specific connectivity of the human homologs, which is their interconnectedness with each other relative to all molecules they are connected to in Ingenuity's Knowledge Database. Networks are limited to 35 molecules each to keep them to a functional size and a network score is generated based on the hypergeometric distribution and is calculated with the right-tailed Fisher's Exact Test. In this study, only the top network for the consolidated gene sets P(H)AHs (P-value = 1.00×10^−39^) and ECs (P-value = 1.00×10^−57^) were used for further analysis.

## Supporting Information

Figure S1 An overview of the workflow for large-scale predictive and discovery chemical biology using whole-adult zebrafish chemogenomics.
(1.12 MB PDF)

Figure S2 Summary of real-time PCR validated genes for identification of biomarkers in 7 selected targeted tissues (brain, gill, liver, gut, skin, testis and eyes).
(0.38 MB PDF)

Table S1 Array samples and concentration of chemical treatments used in the study.
(0.05 MB PDF)

Table S2 Selection criteria and number of genes selected for discriminatory gene sets used for training of prediction models.
(0.01 MB PDF)

Table S3 Summary of the performance of prediction models trained using Dataset I (Figure 1) and Dataset III (Figure 2).
(0.05 MB PDF)

Table S4 Selected BAP- or DES- responsive genes validated to be significant (P<0.05) in whole fish using real-time PCR.
(0.03 MB PDF)

Table S5 Biological associations of the P(H)AH-deregulated human homologs (68) generated by Ingenuity Pathway Analysis (IPA) software.
(0.05 MB PDF)

Table S6 Biological associations of the EC-deregulated human homologs (70) generated by Ingenuity Pathway Analysis (IPA) software.
(0.05 MB PDF)
