# Supplementary material for: Zebrafish Whole-Adult-Organism Chemogenomics for Large-Scale Predictive and Discovery Chemical Biology
Source: PLoS Genet. 2008 Jul 11;4(7):e1000121. doi: 10.1371/journal.pgen.1000121 (PMC2442223; doi:10.1371/journal.pgen.1000121)
Supplement: Figure S1 — An overview of the workflow for large-scale predictive and discovery chemical biology using whole-adult zebrafish chemogenomics. (1.12 MB PDF) [file pgen.1000121.s001.pdf]

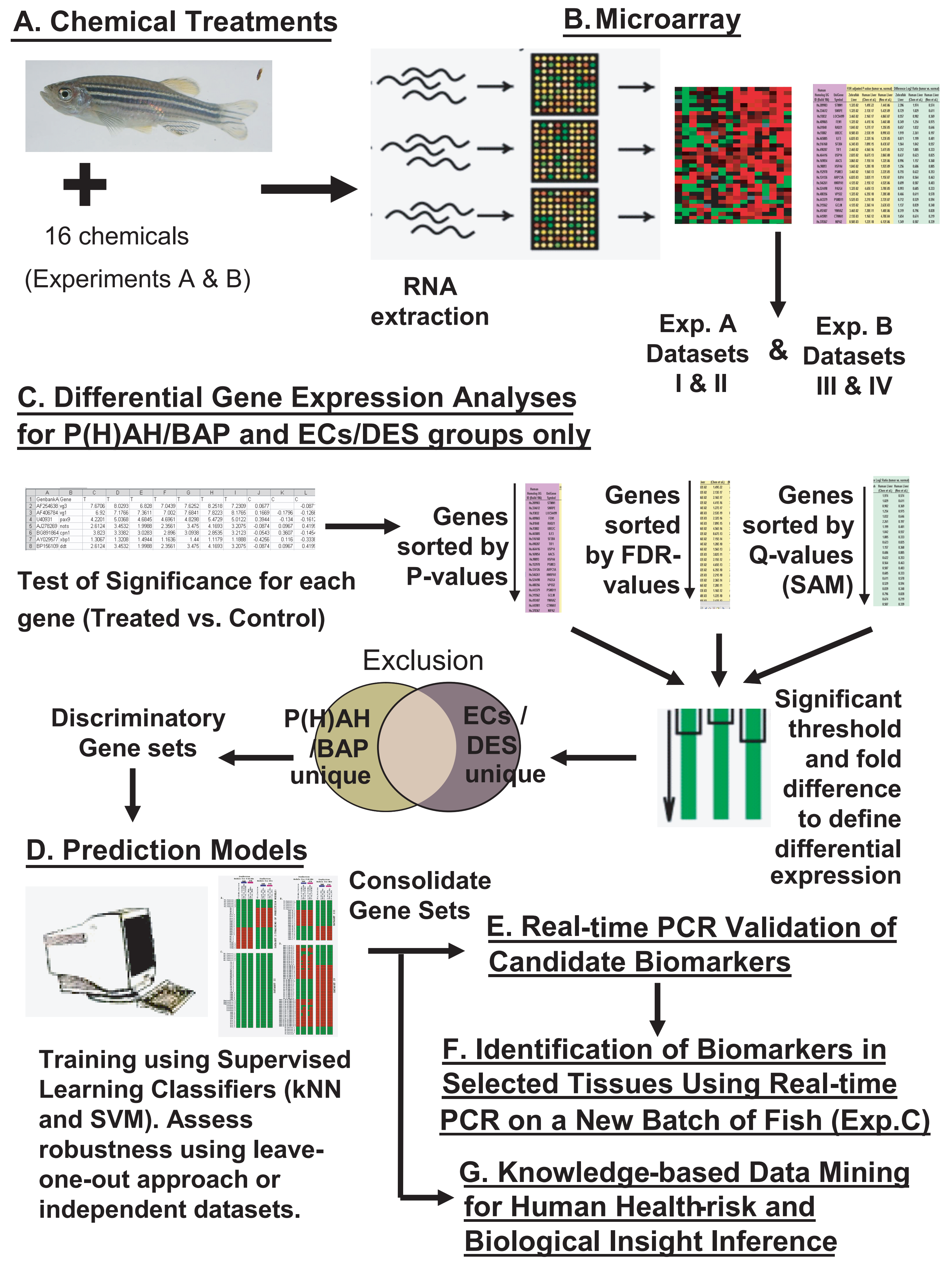

Supplementary Figure 1: An overview of the workflow for large-scale predictive and discovery chemcal biology using whole adult zebrafish chemogenomics.
