# Supplementary material for: Zebrafish Whole-Adult-Organism Chemogenomics for Large-Scale Predictive and Discovery Chemical Biology
Source: PLoS Genet. 2008 Jul 11;4(7):e1000121. doi: 10.1371/journal.pgen.1000121 (PMC2442223; doi:10.1371/journal.pgen.1000121)
Supplement: Figure S2 — Summary of real-time PCR validated genes for identification of biomarkers in 7 selected targeted tissues (brain, gill, liver, gut, skin, testis and eyes). (0.38 MB PDF) [file pgen.1000121.s002.pdf]

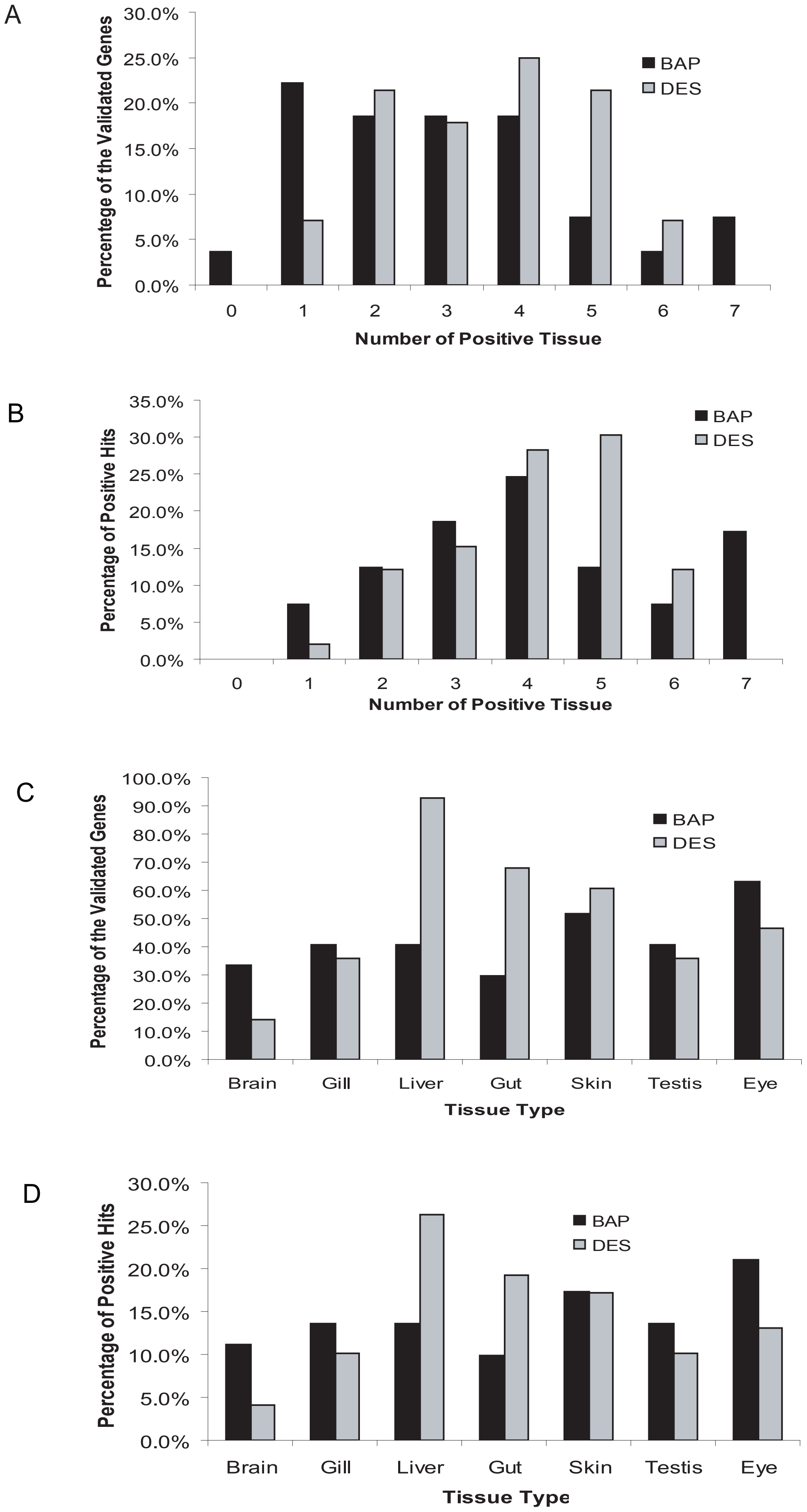

Supplementary Figure 2. Summary of real-time PCR validated genes for identification of biomarkers in 7 selected targeted tissues (brain, gill, liver, gut, skin, testis and eyes). (A) Percentage of the total validated genes [27 genes for Benzo(a)pyrene (BAP) and 28 genes for Diethylstibestrol (DES)] found significantly deregulated in different number of tissue types. (B) Percentage of the total positive hits [i.e. total number of significant gene deregulation (81 for BAP and 99 for DES among the 7 tissues)] in different number of tissue types. (C) Percentage of the total validated genes found significantly deregulated in specific tissue. (D) Percentage of the total positive hits in specific tissue. Statistical significance is determined by heterocedastic Student's T-test (P-value<0.05, n=4) between treatment group versus control (vehicle) group.
