# Supplementary material for: Zebrafish Whole-Adult-Organism Chemogenomics for Large-Scale Predictive and Discovery Chemical Biology
Source: PLoS Genet. 2008 Jul 11;4(7):e1000121. doi: 10.1371/journal.pgen.1000121 (PMC2442223; doi:10.1371/journal.pgen.1000121)
Supplement: Table S1 — Array samples and concentration of chemical treatments used in the study. (0.05 MB PDF) [file pgen.1000121.s003.pdf]

Supplementary Table 1. Array samples and concentration of chemical treatments used in the study

EXPERIMENT A (Expression Chemogenomics)

Group I Samples (Dataset I)

Samples	Treatment	Group
V2.Control_1<br>V2.Control_2<br>V2.Control_3<br>V2.Control_4<br>V2.Control_5	Vehicle alone<br>acetone 0.05%<br>(Merck, Germany)	**Control***
Es_1<br>Es_2<br>Es_3<br>Es_4	17-beta estradiol<br>1ug/L (3.67nM)<br>(Sigma, USA)	**Combined groups of Estrogenic Compounds (ECs)***
Bis_5<br>Bis_1<br>Bis_2<br>Bis_3	Bisphenol A<br>5mg/L (21.9uM)<br>(Aldrich, USA)	**Combined groups of Estrogenic Compounds (ECs)***
DES_3<br>DES_6<br>DES_2<br>DES_8	Diethylstilbestrol<br>1ug/L (3.73nM)<br>(Sigma, USA)	**Combined groups of Estrogenic Compounds (ECs)***
BAP_1<br>BAP_2<br>BAP_3<br>BAP_4<br>BAP_5	Benzo[a]pyrene<br>~5mg/L (~20.0uM)<br>(Sigma, USA)	**Combined groups of Polycyclic (Halogenated) Aromatic Hydrocarbons P(H)AHs***
MC_1<br>MC_2<br>MC_3<br>MC_4	3-Methyl-cholanthrene<br>100ug/L (373nM)<br>(Sigma, USA)	**Combined groups of Polycyclic (Halogenated) Aromatic Hydrocarbons P(H)AHs***
TCDD_1<br>TCDD_2<br>TCDD_3<br>TCDD_5	2,3,7,8-Tetrachloro-dibenzodioxin<br>10ug/L (31.1nM)<br>(CIL, USA)	**Combined groups of Polycyclic (Halogenated) Aromatic Hydrocarbons P(H)AHs***

Group II Samples (Dataset II)

Samples	Treatment	Group
W2.Control_1<br>W2.Control_2<br>W2.Control_3<br>W2.Control_4<br>W2.Control_5	water<br>(control)	
Cd_1<br>Cd_2<br>Cd_3<br>Cd_4<br>Cd_5	Cadmium chloride<br>3mg/L (16.4uM)<br>(Sigma, USA)	Heavy Metals
Hg_1<br>Hg_2<br>Hg_3<br>Hg_4<br>Hg_5	Mercuric chloride<br>0.2mg/L (734nM)<br>(Aldrich, USA)	Heavy Metals
NP_1<br>NP_2<br>NP_3<br>NP_4<br>NP_5	4-Nitrophenol<br>5mg/L (35.9uM)<br>(Merck, Germany)	Organic Nitrogen Compounds
CA_1<br>CA_2<br>CA_3<br>CA_4	4-Chloroaniline<br>20mg/L (157uM)<br>(Merck, Germany)	Organic Nitrogen Compounds
DEN_1<br>DEN_2<br>DEN_3<br>DEN_4<br>DEN_5	Diethylnitrosamine<br>400mg/L (3.9mM)<br>(Sigma, USA)	Organic Nitrogen Compounds
Heptachlor_1<br>Heptachlor_2<br>Heptachlor_3	Heptachlor<br>100ug/l (268nM)<br>(Supelco, USA)	Organochlorine Pesticides
Lindane_1<br>Lindane_2<br>Lindane_3<br>Lindane_5	Lindane<br>100ug/l (344nM)<br>(Aldrich, USA)	Organochlorine Pesticides
DDT_1<br>DDT_2<br>DDT_3<br>DDT_4<br>DDT_5	DDT<br>100ug/l (282nM)<br>(Supelco, USA)	Organochlorine Pesticides
Atz_1<br>Atz_2<br>Atz_3<br>Atz_4<br>Atz_5	Atrazine<br>5mg/L (23.1uM)<br>(Supelco, USA)	Organochlorine Pesticides

* Experimental groups that were used to obtain descriminatory gene sets for training of prediction models (dataset I).

Supplementary Table 1 (continued).

EXPERIMENT B (Expression Chemogenomics)

Group III Samples (Dataset III)

Samples	Treatment	Group
V.Control_1<br>V.Control_10<br>V.Control_5<br>V.Control_7<br>V.Control_8<br>V.Control_9	Vehicle alone<br>acetone 0.01%	**(Control)***
BAP-H_2<br>BAP-H_3<br>BAP-H_4<br>BAP-H_6<br>BAP-H_7	BAP-H: ~500ug/L (~2000nM)	**Combined groups of Benzo[a]pyrene (BAP) ***
BAP-L_1<br>BAP-L_2<br>BAP-L_3<br>BAP-L_6<br>BAP-L_8	BAP-L: ~50ug/L (~200nM)	**Combined groups of Benzo[a]pyrene (BAP) ***
DES-H_1<br>DES-H_4<br>DES-H_5<br>DES-H_9	DES-H: 10 ug/L (37.3nM)	**Combined groups of Diethylstilbestrol (DES) ***
DES-L_3<br>DES-L_4<br>DES-L_5<br>DES-L_6<br>DES-L_7	DES-L: 1 ug/L (3.73nM)	**Combined groups of Diethylstilbestrol (DES) ***

Group IV (Dataset IV)

Samples	Treatment
BAP-L_As-H_1<br>BAP-L_As-H_2<br>BAP-L_As-H_3<br>BAP-L_As-H_5<br>BAP-L_As-H_7<br>BAP-L_As-H_8	BAP-L: ~50ug/L (~200nM)<br>As-H (Arsenic): 40mg/L (~4000nM)<br>(Arsenic purchased from Sigma, USA)
BAP-H_As-H_1<br>BAP-H_As-H_2<br>BAP-H_As-H_3<br>BAP-H_As-H_4<br>BAP-H_As-H_6<br>BAP-H_As-H_7<br>BAP-H_As-H_8	BAP-H: ~500ug/L (~2000nM)<br>As-H (Arsenic): 40mg/L (~4000nM)
BAP-H_DES-H_1<br>BAP-H_DES-H_4<br>BAP-H_DES-H_6	BAP-H: ~500ug/L (~2000nM)<br>DES-H: 10 ug/L (37.3nM)
BAP-H_DES-L_1<br>BAP-H_DES-L_4<br>BAP-H_DES-L_5<br>BAP-H_DES-L_6<br>BAP-H_DES-L_7<br>BAP-H_DES-L_9	BAP-H: ~500ug/L (~2000nM)<br>DES-L: 1 ug/L (3.73nM)

Group IV Samples (Dataset IV) [continued]

Samples	Treatment
As-H_DES-H_1<br>As-H_DES-H_10<br>As-H_DES-H_3<br>As-H_DES-H_5<br>As-H_DES-H_6<br>As-H_DES-H_7<br>As-H_DES-H_8	As-H (Arsenic): 40mg/L (~4000nM)<br>DES-H: 10 ug/L (37.3nM)
As-L_DES-H_3<br>As-L_DES-H_4<br>As-L_DES-H_5<br>As-L_DES-H_6<br>As-L_DES-H_7<br>As-L_DES-H_8	As-L (Arsenic): 4mg/L (~400nM)<br>DES-H: 10 ug/L (37.3nM)
BAP-L_AS-H_DES-H_1<br>BAP-L_AS-H_DES-H_2<br>BAP-L_AS-H_DES-H_5<br>BAP-L_As-H_DES-H_3<br>BAP-L_As-H_DES-H_6<br>BAP-L_As-H_DES-H_7<br>BAP-L_As-H_DES-H_9	BAP-L: ~50ug/L (~200nM)<br>As-H (Arsenic): 40mg/L (~4000nM)<br>DES-H: 10 ug/L (37.3nM)
BAP-L_As-L_DES-H_1<br>BAP-L_As-L_DES-H_4<br>BAP-L_As-L_DES-H_5<br>BAP-L_As-L_DES-H_6<br>BAP-L_As-L_DES-H_7<br>BAP-L_As-L_DES-H_8	BAP-L: ~50ug/L (~200nM)<br>As-L (Arsenic): 4mg/L (~400nM)<br>DES-H: 10 ug/L (37.3nM)
BAP-H_As-H_DES-H_2<br>BAP-H_As-H_DES-H_3<br>BAP-H_As-H_DES-H_6<br>BAP-H_As-H_DES-H_8<br>BAP-H_As-H_DES-H_9	BAP-H: ~500ug/L (~2000nM)<br>As-H (Arsenic): 40mg/L (~4000nM)<br>DES-H: 10 ug/L (37.3nM)
W.Control_1<br>W.Control_2<br>W.Control_4<br>W.Control_5<br>W.Control_7	water<br>(control)

* Experimental groups that were used to obtain descriminatory gene sets for training of prediction models (dataset III).

Supplementary Table 1 (continued).

EXPERIMENT C (Tissue-Specific Biomarker Validation Experiment)

Tissue-specific Samples (brain, gills, liver, gut, skin, testis and eyes were sampled)

Sample	Treatment
V.Control_1	Vehicle alone (Control) acetone 0.01%
V.Control_2	
V.Control_3	
V.Control_4	
V.Control_5	
BAP_1	BAP: ~250ug/L (~1000nM)
BAP_2	
BAP_3	
BAP_4	
BAP_5	
DES_1	DES: 5 ug/L (18.7nM)
DES_2	
DES_3	
DES_4	
DES_5	

Except for 17-beta-estradiol and diethylstilbestrol which was selected based on vitellogenin transcript induction, the exposure concentrations for the remaining compounds were selected based on preliminary acute toxicity tests that produced about 10%-20% mortality within 96 hours of treatment. Concentrations for benzo[a]pyrene were reduced 10- to 100-fold for experiment B and C. We reason that these concentrations would produce a spectrum of responses/effects ranging from adaptive to toxicity.
