# Supplementary material for: Zebrafish Whole-Adult-Organism Chemogenomics for Large-Scale Predictive and Discovery Chemical Biology
Source: PLoS Genet. 2008 Jul 11;4(7):e1000121. doi: 10.1371/journal.pgen.1000121 (PMC2442223; doi:10.1371/journal.pgen.1000121)
Supplement: Table S2 — Selection criteria and number of genes selected for discriminatory gene sets used for training of prediction models. (0.01 MB PDF) [file pgen.1000121.s004.pdf]

Supplementary Table 2. Selection Criteria And Number of Genes Selected For Discriminatory Gene Sets Used For Training of Prediction Models.

Discriminatory Gene Sets	Criteria For Gene Selection*	Number of Selected Genes
**Discriminatory Gene Sets Selected From Dataset I For Training**		
Q -val_P(H)AHs	fold-difference>1.5, *Q* -value<1% in P(H)AH group, *Q* -value>20% in EC group	70
Q -val_ECs	fold-difference>1.5, *Q* -value<1% in EC group, *Q* -value>20% in P(H)AH group	51
FDR -val_P(H)AHs	fold-difference>1.5, *FDR* -value<0.025 in P(H)AH group, *FDR* -value>0.2 in EC group	49
FDR -val_ECs	fold-difference>1.5, *FDR* -value<0.025 in EC, *FDR*-value>0.2 in P(H)AH group	57
P -val_P(H)AHs	fold-difference>1.5, *P* -value<0.0001 in P(H)AH group, *P* -value>0.01 in EC group	42
P -val_ECs	fold-difference>1.5, *P* -value<0.0001 in EC group, *P* -value>0.01 in P(H)AH group	34
**Discriminatory Gene Sets Selected From Dataset III For Training**		
Q -val_P(H)AHs	fold-difference>1.5, *Q* -value<1% in BAP_Low or BAP_High, *Q* -value>20% in DES_High and DES_Low	165
Q -val_ECs	fold-difference>1.5, *Q* -value<1% in DES_Low or DES_High, *Q* -value>20% in BAP_High and BAP_Low	161
FDR -val_P(H)AHs	fold-difference>1.5, *FDR* -value<0.025 in BAP_Low or BAP_High, *FDR* -value>0.2 in DES_High and DES_Low	24
FDR -val_ECs	fold-difference>1.5, *FDR* -value<0.025 in DES_Low or DES_High, *FDR* -value>0.2 in BAP_High and BAP_Low	15
P -val_P(H)AHs	fold-difference>1.5, *P* -value<0.0001 in BAP_Low or BAP_High, *P* -value>0.01 in DES_High and DES_Low	26
P -val_ECs	fold-difference>1.5, *P* -value<0.0001 in DES_Low or DES_High, *P* -value>0.01 in BAP_High and BAP_Low	40

*The stringent criteria is used to ensure high confidence of discriminatory power within a relatively small number of selected genes, although this will likely exclude many other responsive genes.
