# Supplementary material for: Zebrafish Whole-Adult-Organism Chemogenomics for Large-Scale Predictive and Discovery Chemical Biology
Source: PLoS Genet. 2008 Jul 11;4(7):e1000121. doi: 10.1371/journal.pgen.1000121 (PMC2442223; doi:10.1371/journal.pgen.1000121)
Supplement: Table S3 — Summary of the performance of prediction models trained using Dataset I (Figure 1) and Dataset III (Figure 2). (0.05 MB PDF) [file pgen.1000121.s005.pdf]

Supplementary Table 3. Summary of the Performance of Prediction Models Trained Using Dataset I (Figure 1) and Dataset III (Figure 2)

	Prediction Models for P(H)AHs						Prediction Models for ECs					
	kNN			SVM			kNN			SVM		
	Q-val	FDR-val	P-val	Q-val	FDR-val	P-val	Q-val	FDR-val	P-val	Q-val	FDR-val	P-val
Leave-one-out validation on Dataset I [also used as Training Set (Figure 1B)]												
True Positive (TP)	13	13	13	13	13	13	12	12	12	12	12	12
False Positive (FP)	0	0	0	0	0	0	0	0	0	0	0	0
True Negative (TN)	17	17	17	17	17	17	18	18	18	18	18	18
False Negative (FN)	0	0	0	0	0	0	0	0	0	0	0	0
Total	30	30	30	30	30	30	30	30	30	30	30	30
Specificity (%) [TN/(TN+FP)]*	100	100	100	100	100	100	100	100	100	100	100	100
Sensitivity (%) [TP/(TP+FN)]**	100	100	100	100	100	100	100	100	100	100	100	100
Fisher's Exact Test [P-value]	8.35E-09	8.35E-09	8.35E-09	8.35E-09	8.35E-09	8.35E-09	1.16E-08	1.16E-08	1.16E-08	1.16E-08	1.16E-08	1.16E-08
Independent Validation on Dataset II and III [using Dataset I as training set (Figure 1C and D)]												
True Positive (TP)	10	10	10	10	10	10	9	9	9	9	9	9
False Positive (FP)	0	0	0	0	0	0	0	0	0	0	0	0
True Negative (TN)	61	61	61	61	61	61	62	62	62	62	62	62
False Negative (FN)	0	0	0	0	0	0	0	0	0	0	0	0
Total	71	71	71	71	71	71	71	71	71	71	71	71
Specificity (%) [TN/(TN+FP)]	100	100	100	100	100	100	100	100	100	100	100	100
Sensitivity (%) [TP/(TP+FN)]	100	100	100	100	100	100	100	100	100	100	100	100
Fisher's Exact Test [P-value]	2.17E-12	2.17E-12	2.17E-12	2.17E-12	2.17E-12	2.17E-12	1.34E-11	1.34E-11	1.34E-11	1.34E-11	1.34E-11	1.34E-11
Independent Validation on Dataset IV [using Dataset I as training set (Figure 1E)]												
True Positive (TP)	27	34	35	25	37	36	40	40	40	40	40	40
False Positive (FP)	0	0	0	0	0	0	0	0	0	0	0	0
True Negative (TN)	18	18	18	18	18	18	18	18	18	18	18	18
False Negative (FN)	13	6	5	15	3	4	0	0	0	0	0	0
Total	58	58	58	58	58	58	58	58	58	58	58	58
Specificity (%) [TN/(TN+FP)]	100	100	100	100	100	100	100	100	100	100	100	100
Sensitivity (%) [TP/(TP+FN)]	67.5	85	87.5	62.5	92.5	90	100	100	100	100	100	100
Fisher's Exact Test [P-value]	4.69E-07	2.99E-10	7.48E-11	2.39E-06	2.96E-12	1.63E-11	2.22E-15	2.22E-15	2.22E-15	2.22E-15	2.22E-15	2.22E-15

Specificity (%) [TN/(TN+FP)]* is the proportion of true negatives of all negative cases of the samples tested and it measures how well the 'classification test' correctly identifies negative cases.

Sensitivity (%) [TP/(TP+FN)]** is the proportion of true positives of all positive cases of the samples tested and it measures how well the 'classification test' correctly identifies positive cases.

Supplementary Table 3. (Continued)

	Prediction Models for P(H)AHs						Prediction Models for ECs					
	kNN			SVM			kNN			SVM		
	Q-val	FDR-val	P-val	Q-val	FDR-val	P-val	Q-val	FDR-val	P-val	Q-val	FDR-val	P-val
Leave-one-out validation on Dataset III [also used as Training Set (Figure 2B)]												
True Positive (TP)	10	10	10	10	10	10	9	9	9	9	9	9
False Positive (FP)	0	0	0	0	0	0	0	0	0	0	0	0
True Negative (TN)	15	15	15	15	15	15	16	16	16	16	16	16
False Negative (FN)	0	0	0	0	0	0	0	0	0	0	0	0
Total	25	25	25	25	25	25	25	25	25	25	25	25
Specificity (%) [TN/(TN+FP)]*	100	100	100	100	100	100	100	100	100	100	100	100
Sensitivity (%) [TP/(TP+FN)]**	100	100	100	100	100	100	100	100	100	100	100	100
Fisher's Exact Test [P-value]	3.06E-07	3.06E-07	3.06E-07	3.06E-07	3.06E-07	3.06E-07	4.89E-07	4.89E-07	4.89E-07	4.89E-07	4.89E-07	4.89E-07
Independent Validation on Dataset I and II [using Dataset III as training set (Figure 2C and D)]												
True Positive (TP)	13	13	13	13	13	13	10	11	11	10	11	11
False Positive (FP)	1	1	1	1	1	1	0	0	0	0	0	0
True Negative (TN)	62	62	62	62	62	62	64	64	64	64	64	64
False Negative (FN)	0	0	0	0	0	0	2	1	1	2	1	1
Total	76	76	76	76	76	76	76	76	76	76	76	76
Specificity (%) [TN/(TN+FP)]	98.4	98.4	98.4	98.4	98.4	98.4	100	100	100	100	100	100
Sensitivity (%) [TP/(TP+FN)]	100	100	100	100	100	100	83.3	91.7	91.7	83.3	91.7	91.7
Fisher's Exact Test [P-value]	9.17E-14	9.17E-14	9.17E-14	9.17E-14	9.17E-14	9.17E-14	6.91E-11	2.10E-12	2.10E-12	6.91E-11	2.10E-12	2.10E-12
Independent Validation on Dataset IV [using Dataset III as training set (Figure 2E)]												
True Positive (TP)	38	38	36	39	37	37	40	40	40	40	40	40
False Positive (FP)	1	0	0	0	0	0	0	0	0	0	0	0
True Negative (TN)	17	18	18	18	18	18	18	18	18	18	18	18
False Negative (FN)	2	2	4	1	3	3	0	0	0	0	0	0
Total	58	58	58	58	58	58	58	58	58	58	58	58
Specificity (%) [TN/(TN+FP)]	94.4	100	100	100	100	100	100	100	100	100	100	100
Sensitivity (%) [TP/(TP+FN)]	95	95	90	97.5	92.5	92.5	100	100	100	100	100	100
Fisher's Exact Test [P-value]	1.49E-11	4.22E-13	1.63E-11	4.22E-14	2.96E-12	2.96E-12	2.22E-15	2.22E-15	2.22E-15	2.22E-15	2.22E-15	2.22E-15

Specificity (%) [TN/(TN+FP)]* is the proportion of true negatives of all negative cases of the samples tested and it measures how well the 'classification test' correctly identifies negative cases.

Sensitivity (%) [TP/(TP+FN)]** is the proportion of true positives of all positive cases of the samples tested and it measures how well the 'classification test' correctly identifies positive cases.
