# Supplementary material for: Zebrafish Whole-Adult-Organism Chemogenomics for Large-Scale Predictive and Discovery Chemical Biology
Source: PLoS Genet. 2008 Jul 11;4(7):e1000121. doi: 10.1371/journal.pgen.1000121 (PMC2442223; doi:10.1371/journal.pgen.1000121)
Supplement: Table S4 — Selected BAP- or DES- responsive genes validated to be significant (P<0.05) in whole fish using real-time PCR. (0.03 MB PDF) [file pgen.1000121.s006.pdf]

Supplementary Table 4. Selected BAP- or DES- responsive genes validated to be significant (*P* <0.05) in whole fish using real-time PCR.

Genbank Acc	Descpription	Gene Symbol	Log 2 Fold Difference (Microarray)	***Log 2 Fold Difference (PCR)
BAP-responsive genes in P(H)AH Discriminatory Gene Set				
AW342687	Cytochrome P450, family 1, subfamily A *	cyp1A	4.06	5.44
AF057713	Cytochrome P450, family 1, subfamily A, polypeptide1 *	cyp1A1	3.90	3.97
BM183152	CDNA clone IMAGE:7177046		1.41	2.82
BG303549	Carbonic anhydrase II	ca2	1.31	2.06
BI430015	Zgc:92102	zgc:92102	1.28	2.36
AI353694	GIF homolog	gif	1.26	1.63
BG306468	TIPARP homolog *	TIPARP	1.24	1.97
AI959735	SULT6B1 homolog	SULTB1	0.98	2.35
AF285098	Glutathione S-transferase pi *	gstp1	0.90	1.00
AI601682	CYB5A homolog	cyb5a	0.74	1.64
BI891596	NAD(P)H dehydrogenase, quinone 1 *	nqo1	0.72	1.49
AI477969	GRHL3 homolog	grhl3	0.63	0.96
AW232207	Transcribed locus		0.62	3.17
AF063446	Aryl hydrocarbon receptor 2 *	ahr2	0.62	1.41
AI667240	Carboxypeptidase B1	cpb1	-0.64	-1.03
AI397347	KRT17 homolog	krt17	-1.30	-0.81
BAP-responsive genes in Non-P(H)AH Discriminatory Gene Set				
BM025955	Transcribed locus		1.31	3.23
BG304178	Transcribed locus		0.71	0.49
AI793886	Wu:fc55f04	wu:fc55f04	0.40	0.34
AW232794	Sortilin 1	sort1	0.60	1.31
BI878941	Zgc:77849	zgc:77849	0.56	1.15
AW018635	Transforming growth factor, beta-inducec	tgfbi	-0.46	-1.30
AW233556	Wu:fj37e01	Wu:fj37e01	-0.59	-0.33
AF295407	Alcohol dehydrogenase 8a	adh8a	-0.79	-1.57
BG304220	Wu:fl33b06	wu:fl33b06	-1.12	-1.18
AF064835	Eukaryotic translation elongation factor 2, like	eef2l	-1.16	-1.12
AW421939	Hm:zeh1207	Hm:zeh1207	-1.52	-1.07
DES-responsive genes in EC Discriminatory Gene Set				
AF406784	vitellogenin 1 **	vg1	6.07	12.3
AF254638	Vitellogenin 3, phosvitinless **	vg3	5.88	12.87
BI427744	FK506 binding protein 11 **	fkbp11	2.68	3.31
BG891864	Carboxypeptidase N, polypeptide 1	cpn1	2.51	2.88
BI839255	Wu:fb78c04	wu:fb78c04	2.23	0.59
BM082666	Zgc:92744	zgc:92744	2.09	1.97
AI522628	Wu:fb60e09	wu:fb60e09	1.86	1.74
BI889078	High density lipoprotein-binding protein (vigilin)	hdlbp	1.39	1.31
AW175541	Wu:fi36e01	wu:fi36e01	1.33	2.29
BF717944	Transcribed locus, moderately similar to XP_416256.1		1.28	4.74
BF717555	nuclear protein 1	nupr1	1.20	3.52
BI885968	Signal sequence receptor, delta	ssr4	1.17	1.37
BI882727	Signal sequence receptor, alpha	ssr1	1.16	1.19
BM182319	Solute carrier family 31 (copper transporters), member 1	slc31a1	1.10	1.40
AF349412	Estrogen receptor 1 **	esr1	0.79	4.60
BI896378	Zgc:66313	zgc:66313	0.58	1.65
BF717503	Serine (or cysteine) proteinase inhibitor, clade C member 1	serpinc1	-0.61	-0.89
AW342687	Cytochrome P450, family 1, subfamily A *	cyp1A	-0.77	-0.95
BF156623	Uncoupling protein 4	ucp4	-1.12	-1.83
AW019201	Complement Component 9	c9	-2.03	-2.35
AW115757	Hemopexin		-3.37	-3.23
DES-responsive genes in Non-ECs Discriminatory Gene Set				
BI878941	Zgc:77849	zgc:77849	0.86	0.73
BI981380	Wu:fj36g07	wu:fj36g07	0.67	0.38
AF057713	Cytochrome P450, family 1, subfamily A, polypeptide1	cyp1A1	-0.35	-0.65
AW421213	Phenylalanine hydroxylase	pah	-0.95	-0.68
BG985468	Fructose-1,6-bisphosphatase 1	fbp1	-1.08	-2.09
AI722510	MGC:103610 IMAGE:7250917		-1.38	-2.11
AW019124	Alanine-glyoxylate aminotransferase	agxt	-1.42	-1.47

* Known BAP-responsive genes.

** Known Estrogen-responsive genes.

*** All values shown are significant (*P* -value<0.05; n=4-5) by heterocedastic T-Test when compared to their respective control group.
