# Supplementary material for: Zebrafish Whole-Adult-Organism Chemogenomics for Large-Scale Predictive and Discovery Chemical Biology
Source: PLoS Genet. 2008 Jul 11;4(7):e1000121. doi: 10.1371/journal.pgen.1000121 (PMC2442223; doi:10.1371/journal.pgen.1000121)
Supplement: Table S5 — Biological associations of the P(H)AH-deregulated human homologs (68) generated by Ingenuity Pathway Analysis (IPA) software. (0.05 MB PDF) [file pgen.1000121.s007.pdf]

Supplementary Table 5. Biological associations of the P(H)AH-deregulated human homologs (68) generated by Ingenuity Pathway Analysis (IPA) software.

Description of Biological Association	P-value	Molecules (human homologs) Involved		
		Number	Percentage	*Gene Symbol
**Molecular and Cellular Functions**				
Cell Signaling	1.29E-07-8.41E-03	31	45.6%	ACTG1,SULT2B1,CYP1A1,ATRNL1,RAC1,EIF2S2,CXCL14,PPP3R2,LPL,MMP13,HSP90AB1,RPS6KA1,NOS2A,IGF1,CYP2C19,RHOT1,ALAS1,PSMA3,NQO1,ANXA1,CYP1B1,GSTP1,ELF3,ARHGEF1,NR3C1,STAT1,MAL,CTBP1,NFE2L2,PLP1,AHR
Cell Death	1.05E-04-9.87E-03	31	45.6%	RAC1,FOSL2,MMP13,HSP90AB1,RPS6KA1,ODC1,NOS2A,CDK8,IGF1,RHOT1,CYB5A,NQO1,PPM1D,ANXA1,SCIN,AGPAT2,MTMR1,CYP1B1,FABP1,GSTP1,ABCG2,ELF3,WWOX,NR3C1,STAT1,MAL,CTBP1,NFE2L2,PLP1,AHR,NP
Small Molecule Biochemistry	4.64E-07-9.87E-03	25	36.8%	CYP1A1,SULT2B1,RAC1,THRSP,LPL,NOS2A,ODC1,IGF1,CYP2C19,TNXB,CYB5A,NQO1,ANXA1,CPA1,AGPAT2,FABP1,CYP1B1,GSTP1,ABCG2,SLC3A2,WWOX,NR3C1,PLP1,AHR,NP
Cellular Growth and Proliferation	1.8E-04-9.75E-03	23	33.8%	ACTG1,CYP1A1,ANXA11,RAC1,FOSL2,VAMP8,RPS6KA1,FABP1,ODC1,GSTP1,NOS2A,SLC3A2,ELF3,IGF1,NR3C1,ARHGEF1,STAT1,CTBP1,RSL1D1,PLP1,PPM1D,ANXA1,SCAMP2
Lipid Metabolism	9.58E-06-9.63E-03	21	30.9%	SULT2B1,CYP1A1,RAC1,AGPAT2,THRSP,LPL,CYP1B1,FABP1,NOS2A,GSTP1,WWOX,SLC3A2,IGF1,ABCG2,NR3C1,CYP2C19,TNXB,PLP1,AHR,NQO1,ANXA1
Gene Expression	9.87E-05-9.87E-03	17	25.0%	CYP1A1,ZNF238,RAC1,FOSL2,THRSP,FLII,WWOX,CDK8,ELF3,IGF1,NR3C1,STAT1,CTBP1,ALAS1,NFE2L2,AHR,CYB5A
Cellular Development	6.63E-04-9.31E-03	17	25.0%	SCIN,FOSL2,MMP13,RPS6KA1,NOS2A,ODC1,ABCG2,IGF1,ELF3,NR3C1,STAT1,HOXA2,MAL,CTBP1,PLP1,NP,ANXA1
Cellular Movement	1.68E-04-8.96E-03	15	22.1%	ACTG1,RAC1,FOSL2,CXCL14,MMP13,HSP90AB1,NOS2A,IGF1,SLC3A2,ELF3,ARHGEF1,STAT1,NFE2L2,PLP1,ANXA1
Cell Cycle	1.17E-03-9.82E-03	14	20.6%	RAC1,FOSL2,FABP1,IGF1,SLC3A2,CDK8,WWOX,KIFC1,NR3C1,ARHGEF1,STAT1,AHR,PPM1D,ANXA1
Molecular Transport	1.54E-03-9.63E-03	14	20.6%	LPL,CYP1B1,FABP1,ODC1,NOS2A,ABCG2,IGF1,SLC3A2,WWOX,NR3C1,PLP1,AHR,NQO1,NP
Drug Metabolism	1.29E-07-5.72E-03	12	17.6%	CYP1A1,SULT2B1,ABCG2,CYP2C19,ALAS1,NFE2L2,HSP90AB1,AHR,CYP1B1,NQO1,NOS2A,GSTP1
Cellular Assembly and Organization	1.92E-04-8.41E-03	11	16.2%	SLC3A2,IGF1,KIFC1,ARHGEF1,CTBP1,TNXB,RAC1,CLINT1,PLP1,ANXA1,SCAMP2
Cellular Function and Maintenance	1.92E-04-8.6E-03	11	16.2%	SLC3A2,ABCG2,IGF1,NR3C1,ARHGEF1,STAT1,RAC1,LPHN2,NQO1,NOS2A,SCAMP2
Cell-To-Cell Signaling and Interaction	6.64E-04-9.87E-03	8	11.8%	ABCG2,IGF1,STAT1,RAC1,MMP13,NFE2L2,AHR,NOS2A
Cellular Compromise	1.06E-03-4.95E-03	8	11.8%	ACTG1,IGF1,SCIN,CTBP1,NFE2L2,NQO1,GSTP1,FLII
Cell Morphology	1.06E-03-9.61E-03	7	10.3%	IGF1,ARHGEF1,SCIN,RAC1,PLP1,AHR,ODC1
Nucleic Acid Metabolism	1.06E-03-9.61E-03	6	8.8%	CYP1A1,GDA,CYP2C19,CYP1B1,NQO1,NP
Vitamin and Mineral Metabolism	9.58E-06-4.95E-03	4	5.9%	CYP1A1,CYP2C19,LPL,CYP1B1
Amino Acid Metabolism	1.54E-03-6.74E-03	4	5.9%	SLC3A2,IGF1,CPA1,NR3C1
Energy Production	4.83E-03-4.95E-03	4	5.9%	WWOX,PC,CYP1B1,NOS2A
DNA Replication, Recombination, and Repair	4.95E-03-4.95E-03	2	2.9%	KIFC1,NR3C1
Free Radical Scavenging	4.95E-03-4.95E-03	1	1.5%	RAC1
Post-Translational Modification	4.95E-03-4.95E-03	1	1.5%	IGF1
Protein Degradation	4.95E-03-4.95E-03	1	1.5%	LPL
Protein Synthesis	4.95E-03-4.95E-03	1	1.5%	IGF1
RNA Damage and Repair	4.95E-03-4.95E-03	1	1.5%	NOS2A
RNA Post-Transcriptional Modification	4.95E-03-4.95E-03	1	1.5%	NOS2A

* Gene symbol in red or green lettering indicates that the gene is up- or down-regulated, respectively.

Supplementary Table 5. (Continued)

Description of Biological Association	P-value	Molecules (human homologs) Involved		
		Number	Percentage	*Gene Symbol
**Physiological System Development and Function**				
Hematological System Development and Function	2.16E-05-8.06E-03	19	27.9%	RAC1,CXCL14,MMP13,FABP1,NOS2A,GSTP1,SLC3A2,IGF1,ABCG2,NR3C1,STAT1,NFE2L2,PLP1,AHR,NQO1,PPM1D,ANXA1,NP,SCAMP2
Tissue Morphology	2.16E-05-9E-03	16	23.5%	RAC1,FOSL2,MMP13,RPS6KA1,FABP1,GSTP1,NOS2A,IGF1,ABCG2,NR3C1,NFE2L2,PLP1,AHR,NQO1,PPM1D,NP
Immune and Lymphatic System Development and Function	2.64E-05-5.24E-03	15	22.1%	SCIN,MMP13,RPS6KA1,FABP1,NOS2A,GSTP1,IGF1,ABCG2,NR3C1,CTBP1,PLP1,AHR,NQO1,PPM1D,NP
Connective Tissue Development and Function	6.64E-04-9.46E-03	12	17.6%	IGF1,NR3C1,HOXA2,STAT1,RAC1,FOSL2,MMP13,AHR,NQO1,GSTP1,PPM1D,NOS2A
Tissue Development	8.44E-05-9.61E-03	11	16.2%	IGF1,NR3C1,HOXA2,STAT1,CTBP1,RAC1,FOSL2,MMP13,FLII,GRHL3,NOS2A
Endocrine System Development and Function	2.42E-05-5.72E-03	10	14.7%	CYP1A1,SULT2B1,ABCG2,IGF1,CYP2C19,LPHN2,AHR,RPS6KA1,CYP1B1,GSTP1
Skeletal and Muscular System Development and Function	2.39E-04-9.75E-03	9	13.2%	IGF1,NR3C1,HOXA2,STAT1,RAC1,FOSL2,MMP13,AHR,NOS2A
Organismal Development	6.62E-05-7.27E-03	8	11.8%	IGF1,NR3C1,CTBP1,MMP13,RPS6KA1,PPM1D,NOS2A,ODC1
Immune Response	1.95E-03-8.06E-03	8	11.8%	IGF1,RAC1,CXCL14,NFE2L2,PLP1,NOS2A,ANXA1,SCAMP2
Organ Development	2.89E-05-6.74E-03	7	10.3%	IGF1,NR3C1,FOSL2,CXCL14,CYP1B1,NOS2A,ODC1
Visual System Development and Function	2.89E-05-9.87E-03	6	8.8%	IGF1,NR3C1,FOSL2,CXCL14,CYP1B1,NOS2A
Nervous System Development and Function	3.58E-04-9.87E-03	6	8.8%	IGF1,NR3C1,STAT1,RAC1,PLP1,NOS2A
Cardiovascular System Development and Function	3.51E-03-6.74E-03	6	8.8%	IGF1,LPL,MMP13,AHR,NOS2A,ODC1
Embryonic Development	4.08E-03-4.95E-03	6	8.8%	SLC3A2,IGF1,RAC1,FOSL2,AHR,FLII
Organ Morphology	4.95E-03-9.61E-03	6	8.8%	IGF1,NR3C1,TNXB,TECTA,AHR,NOS2A
Reproductive System Development and Function	1.68E-04-9.61E-03	4	5.9%	ELF3,IGF1,RAC1,NOS2A
Hepatic System Development and Function	6.64E-04-3.51E-03	3	4.4%	IGF1,STAT1,MMP13
Auditory and Vestibular System Development and Function	4.95E-03-4.95E-03	2	2.9%	IGF1,TECTA
Hair and Skin Development and Function	4.95E-03-4.95E-03	2	2.9%	IGF1,TNXB
Digestive System Development and Function	4.95E-03-4.95E-03	1	1.5%	IGF1
Renal and Urological System Development and Function	4.95E-03-4.95E-03	1	1.5%	IGF1
Tumor Morphology	4.95E-03-4.95E-03	1	1.5%	IGF1

* Gene symbol in red or green lettering indicates that the gene is up- or down-regulated, respectively.

Supplementary Table 5. (Continued)

Description of Biological Association	P-value	Molecules (human homologs) Involved		
		Number	Percentage	*Gene Symbol
**Diseases and Disorders**				
Cancer**	1.98E-05-9.82E-03	34	50.0%	ACTG1,CYP1A1,RAC1,MMP13,HSP90AB1,RPS6KA1,NOS2A,ODC1,IGF1,CDK8,CYP2C19,CYB5A,NQO1,PPM1D,ANXA1,SCIN,AGPAT2,PLCL2,MTMR1,FABP1,CYP1B1,FLII,GSTP1,ABCG2,ELF3,WWOX,SLC3A2,ARHGEF1,NR3C1,STAT1,CTBP1,NFE2L2,AHR,NP
Reproductive System Disease**	1.05E-04-9.75E-03	16	23.5%	RAC1,AGPAT2,MMP13,FABP1,CYP1B1,NOS2A,ODC1,GSTP1,IGF1,CDK8,ABCG2,NR3C1,STAT1,AHR,CYB5A,NQO1
Hematological Disease	8.61E-04-9.43E-03	14	20.6%	CYP1A1,ANXA11,SCIN,RAC1,NOS2A,CDK8,IGF1,NR3C1,STAT1,CTBP1,NFE2L2,NQO1,ANXA1,NP
Inflammatory Disease**	4.99E-04-9.87E-03	13	19.1%	CA7,MMP13,PPP3R2,NOS2A,ODC1,CPA3,IGF1,NR3C1,STAT1,AHNAK,NFE2L2,PLP1,AHR
Genetic Disorder	4.95E-03-6.45E-03	13	19.1%	GLDC,AGPAT2,LPL,MMP13,ODC1,NOS2A,NR3C1,AHNAK,POMGNT1,TECTA,PLP1,AHR,PPM1D
Immunological Disease**	5.02E-04-9.87E-03	12	17.6%	IGF1,NR3C1,STAT1,NFE2L2,PPP3R2,MMP13,PLP1,AHR,NQO1,NOS2A,ANXA1,NP
Organismal Injury and Abnormalities	8.8E-04-9.75E-03	10	14.7%	IGF1,STAT1,RAC1,MMP13,NFE2L2,LPL,AHR,FLII,NOS2A,GSTP1
Endocrine System Disorders**	1.08E-03-7.03E-03	9	13.2%	IGF1,NR3C1,CA7,STAT1,AGPAT2,LPL,AHR,RPS6KA1,NOS2A
Metabolic Disease	2.28E-03-9.03E-03	9	13.2%	IGF1,NR3C1,CA7,GLDC,GIF,AGPAT2,LPL,RPS6KA1,NOS2A
Neurological Disease**	4.99E-04-9.87E-03	8	11.8%	IGF1,NR3C1,STAT1,NFE2L2,POMGNT1,PLP1,AHR,NOS2A
Dermatological Diseases and Conditions	1.25E-03-9.61E-03	8	11.8%	NR3C1,TNXB,RAC1,PPP3R2,AHR,NQO1,GSTP1,ODC1
Connective Tissue Disorders	4.95E-03-9.87E-03	7	10.3%	WWOX,ABCG2,IGF1,RAC1,MMP13,FABP1,GSTP1
Respiratory Disease	1.03E-03-9.87E-03	6	8.8%	WWOX,IGF1,NR3C1,STAT1,NFE2L2,NOS2A
Viral Function	2.39E-04-9E-03	5	7.4%	IGF1,NR3C1,STAT1,RAC1,NOS2A
Developmental Disorder	5.71E-04-4.95E-03	4	5.9%	IGF1,RAC1,AHR,FLII
Ophthalmic Disease	1.06E-03-7.27E-03	4	5.9%	IGF1,NR3C1,CA7,CYP1B1
Cardiovascular Disease	1.25E-03-4.95E-03	4	5.9%	IGF1,LPL,AHR,NOS2A
Hepatic System Disease	2.12E-03-9E-03	4	5.9%	NR3C1,STAT1,MMP13,AHR
Skeletal and Muscular Disorders	3.51E-03-9.87E-03	4	5.9%	IGF1,RAC1,MMP13,POMGNT1
Infectious Disease	6.74E-03-7.27E-03	4	5.9%	NR3C1,STAT1,CA7,NOS2A
Gastrointestinal Disease	4.95E-03-9E-03	3	4.4%	STAT1,MMP13,AHR
Nutritional Disease	5.03E-03-5.03E-03	3	4.4%	NR3C1,LPL,FABP1
Renal and Urological Disease	1.29E-03-1.54E-03	2	2.9%	IGF1,NOS2A
Psychological Disorders	9.87E-03-9.87E-03	1	1.5%	IGF1

* Gene symbol in red or green lettering indicates that the gene is up- or down-regulated, respectively.

**Associated with AHR, NR3C1/GR, STAT1 and IGF1 molecules.
