# Supplementary material for: Zebrafish Whole-Adult-Organism Chemogenomics for Large-Scale Predictive and Discovery Chemical Biology
Source: PLoS Genet. 2008 Jul 11;4(7):e1000121. doi: 10.1371/journal.pgen.1000121 (PMC2442223; doi:10.1371/journal.pgen.1000121)
Supplement: Table S6 — Biological associations of the EC-deregulated human homologs (70) generated by Ingenuity Pathway Analysis (IPA) software. (0.05 MB PDF) [file pgen.1000121.s008.pdf]

Supplementary Table 6. Biological associations of the EC-deregulated human homologs (70) generated by Ingenuity Pathway Analysis (IPA) software.

Description of Biological Association	P-value	Molecules (human homologs) Involved		
		Number	Percentage	*Gene Symbol
**Molecular and Cellular Functions**				
Small Molecule Biochemistry	8.63E-05-4.76E-02	22	31.4%	SERPINA1,APOB,CYP51A1,CYP1A1,MAOB,PGK1,HDLBP,ESR1,CPN1,SC4MOL,PISD,DIO2,CHPT1,NPC2,GPX4,CYP2C19,FKBP4,CDO1,ACSL4,UCP2,XBP1,HPX
Cellular Growth and Proliferation	4.03E-03-4.43E-02	22	31.4%	SERPINA1,CYP1A1,PSMC5,DIABLO,DAP,PGK1,PIM3,ESR1,AHSG,KLF6,PTCH1,GPX4,PA2G4,QSCN6,FKBP4,POSTN,DAB2,UCP2,XBP1,PKP3,P8,TXNL1
Lipid Metabolism	8.63E-05-4.76E-02	16	22.9%	SERPINA1,APOB,CYP51A1,CYP1A1,HDLBP,ESR1,SC4MOL,PISD,CHPT1,NPC2,GPX4,CYP2C19,FKBP4,ACSL4,XBP1,UCP2
Molecular Transport	1.62E-04-4.24E-02	14	20.0%	APOB,SLC22A2,SLC30A7,SLC31A1,ESR1,DIO2,GPX4,FKBP4,CDO1,ACSL4,UCP2,XBP1,TXNL1,HPX
Cellular Assembly and Organization	5.4E-03-4.24E-02	11	15.7%	DNM2,REPS1,TMED10,FKBP4,ANXA13,CLINT1,DAB2,TRIM63,XBP1,SERP1,PTCH1
Gene Expression	5.4E-03-4.76E-02	10	14.3%	PA2G4,GPX4,CYP1A1,CALCOCO1,ESR1,UCP2,DAB2,KLF6,CISH,SND1
Amino Acid Metabolism	5.4E-03-4.24E-02	8	11.4%	CYP51A1,CYP1A1,MAOB,CYP2C19,LDHB,CDO1,CPN1,DIO2
Protein Synthesis	2.07E-02-4.76E-02	8	11.4%	APOB,CYP1A1,PGA5,RPL30,EIF4G3,CPN1,PABPC4,RRBP1
Cellular Function and Maintenance	2.63E-03-4.76E-02	7	10.0%	DNM2,REPS1,FKBP4,CLINT1,ESR1,UCP2,DAB2
Cellular Development	5.4E-03-4.43E-02	7	10.0%	DIABLO,POSTN,ESR1,DAB2,XBP1,P8,PKP3
Drug Metabolism	1.54E-03-4.76E-02	5	7.1%	GPX4,CYP1A1,CYP2C19,PGK1,ESR1
Protein Degradation	3.74E-03-3.74E-03	5	7.1%	PSMB6,PSMC6,PSMC5,PSMC2,PSMD13
Cellular Movement	5.4E-03-4.24E-02	5	7.1%	DNM2,APOB,POSTN,ESR1,PKP3
Vitamin and Mineral Metabolism	1.01E-03-3.2E-02	4	5.7%	NPC2,CYP1A1,CYP2C19,DIO2
Cell Death	5.4E-03-4.76E-02	4	5.7%	APOB,ESR1,UCP2,DAB2
Cell Morphology	5.4E-03-3.2E-02	4	5.7%	TMED10,ESR1,TRIM63,XBP1
Nucleic Acid Metabolism	5.4E-03-3.2E-02	3	4.3%	CYP2C19,PGK1,UCP2
Energy Production	6.17E-03-6.17E-03	3	4.3%	GPX4,CDO1,TXNL1
Cell-To-Cell Signaling and Interaction	9.28E-03-4.24E-02	3	4.3%	POSTN,ESR1,DAB2
Free Radical Scavenging	5.4E-03-5.4E-03	1	1.4%	SERPINA1
Carbohydrate Metabolism	1.08E-02-3.72E-02	1	1.4%	ESR1
Cell Signaling	1.08E-02-1.08E-02	1	1.4%	DIO2
DNA Replication, Recombination, and Repair	1.08E-02-1.08E-02	1	1.4%	PTCH1
Post-Translational Modification	1.08E-02-1.08E-02	1	1.4%	MAOB
Cellular Compromise	2.67E-02-2.67E-02	1	1.4%	HPX
Cell Cycle	4.24E-02-4.24E-02	1	1.4%	ESR1

* Gene symbol in red or green lettering indicates that the gene is up- or down-regulated, respectively.

Supplementary Table 6. (Continued)

Description of Biological Association	P-value	Molecules (human homologs) Involved		
		Number	Percentage	*Gene Symbol
**Physiological System Development and Function**				
Tissue Development	4.37E-03-4.56E-02	8	11.4%	EMILIN1,RBP4,POSTN,ESR1,DAB2,PTCH1,DIO2,CISH
Skeletal and Muscular System Development and Function	1.08E-02-4.76E-02	8	11.4%	DNM2,PAX1,CYP1A1,POSTN,ESR1,AHSG,TRIM63,DIO2
Organismal Development	1.61E-02-4.24E-02	8	11.4%	PAX1,SERPINC1,POSTN,SLC31A1,ESR1,DAB2,SERP1,PTCH1
Organ Development	5.4E-03-4.24E-02	7	10.0%	PAX1,TMED10,ESR1,XBP1,PTCH1,DIO2,CISH
Endocrine System Development and Function	8.63E-05-4.76E-02	6	8.6%	PAX1,CYP1A1,CYP2C19,FKBP4,ESR1,DIO2
Embryonic Development	5.4E-03-3.2E-02	6	8.6%	RBP4,SERPINC1,POSTN,SLC31A1,DAB2,PTCH1
Connective Tissue Development and Function	1.08E-02-4.24E-02	6	8.6%	DNM2,PAX1,POSTN,AHSG,ESR1,PKP3
Reproductive System Development and Function	5.4E-03-4.76E-02	5	7.1%	APOB,ESR1,DAB2,PTCH1,CISH
Tumor Morphology	5.4E-03-4.76E-02	5	7.1%	PGK1,SERPINC1,ESR1,AHSG,PTCH1
Organ Morphology	1.21E-03-4.76E-02	4	5.7%	PAX1,ESR1,XBP1,DIO2
Tissue Morphology	5.4E-03-4.24E-02	4	5.7%	PAX1,APOB,SLC31A1,ESR1
Hair and Skin Development and Function	9.28E-03-4.3E-02	4	5.7%	POSTN,ESR1,DAB2,PTCH1
Renal and Urological System Development and Function	1.79E-02-4.43E-02	4	5.7%	ESR1,PKP3,KLF6,PTCH1
Immune Response	1.37E-02-3.72E-02	3	4.3%	GPX4,ESR1,AHSG
Cardiovascular System Development and Function	2.67E-02-4.76E-02	3	4.3%	SERPINC1,ESR1,DIO2
Auditory and Vestibular System Development and Function	5.4E-03-2.14E-02	2	2.9%	TMED10,DIO2
Nervous System Development and Function	1.08E-02-4.24E-02	2	2.9%	ESR1,DIO2
Hematological System Development and Function	1.61E-02-3.72E-02	2	2.9%	ESR1,XBP1
Digestive System Development and Function	2.67E-02-4.24E-02	2	2.9%	DIABLO,XBP1
Behavior	5.4E-03-4.76E-02	1	1.4%	ESR1
Organismal Survival	5.4E-03-5.4E-03	1	1.4%	SERPINC1
Hepatic System Development and Function	1.61E-02-2.67E-02	1	1.4%	XBP1
Respiratory System Development and Function	1.61E-02-1.61E-02	1	1.4%	ESR1
Immune and Lymphatic System Development and Function	2.67E-02-2.67E-02	1	1.4%	ESR1

* Gene symbol in red or green lettering indicates that the gene is up- or down-regulated, respectively.

Supplementary Table 6. (Continued)

Description of Biological Association	P-value	Molecules (human homologs) Involved		
		Number	Percentage	*Gene Symbol
**Diseases and Disorders**				
Cancer	4.27E-04-4.76E-02	14	20.0%	SERPINA1,CYP1A1,TRAM1,DIABLO,PGK1,ESR1,AHSG,KLF6,PTCH1,SERPINC1,POSTN,DAB2,UCP2,P8
Neurological Disease	1.09E-03-4.76E-02	9	12.9%	TMED10,MAOB,LDHB,PGK1,ESR1,UCP2,RNF10,KLF6,PTCH1
Genetic Disorder	5.4E-03-4.76E-02	9	12.9%	DNM2,SERPINA1,NPC2,APOB,LMAN1,FGB,EDARADD,PTCH1,AMY2B
Psychological Disorders	3.34E-04-3.72E-02	6	8.6%	GPX4,PSMC6,DIABLO,FBXO9,ESR1,XBP1
Hematological Disease	5.4E-03-3.72E-02	6	8.6%	APOB,LMAN1,FGB,SERPINC1,ESR1,HPX
Inflammatory Disease	5.4E-03-4.76E-02	5	7.1%	SERPINA1,FGB,POSTN,ESR1,HPX
Reproductive System Disease	5.4E-03-4.76E-02	5	7.1%	SERPINA1,CYP1A1,ESR1,DAB2,P8
Cardiovascular Disease	1.08E-02-4.76E-02	5	7.1%	APOB,SERPINC1,ESR1,PTCH1,HPX
Organismal Injury and Abnormalities	1.08E-02-3.72E-02	5	7.1%	SERPINA1,APOB,FGB,ESR1,AHSG
Developmental Disorder	1.21E-03-3.72E-02	4	5.7%	PAX1,EDARADD,ESR1,XBP1
Nutritional Disease	6.42E-03-2.56E-02	4	5.7%	APOB,ESR1,UCP2,AMY2B
Hepatic System Disease	1.08E-02-4.76E-02	4	5.7%	SERPINA1,ESR1,XBP1,HPX
Dermatological Diseases and Conditions	1.08E-02-3.72E-02	3	4.3%	SERPINA1,POSTN,PTCH1
Gastrointestinal Disease	1.53E-02-4.76E-02	3	4.3%	ESR1,XBP1,HPX
Viral Function	3.1E-02-4.84E-02	3	4.3%	PA2G4,CALCOCO1,KLF6
Metabolic Disease	5.4E-03-4.76E-02	2	2.9%	NPC2,APOB
Respiratory Disease	1.08E-02-4.24E-02	2	2.9%	SERPINA1,AHSG
Skeletal and Muscular Disorders	1.61E-02-4.76E-02	2	2.9%	DNM2,FGB
Endocrine System Disorders	2.14E-02-3.2E-02	2	2.9%	UCP2,P8
Renal and Urological Disease	2.14E-02-4.24E-02	2	2.9%	CYP1A1,P8
Immunological Disease	3.2E-02-3.72E-02	1	1.4%	PAX1
Connective Tissue Disorders	4.76E-02-4.76E-02	1	1.4%	FGB

* Gene symbol in red or green lettering indicates that the gene is up- or down-regulated, respectively.
